# Artificial intelligence facilitates urban green transition in the Yangtze River Delta urban agglomeration

**DOI:** 10.1038/s41598-026-58535-6

**Published:** 2026-06-17

**Authors:** Weiming Yu, Xiaoyan Yang, Linlin Zheng, Qingshan Wang

**Affiliations:** 1 School of Economics and Management, Beibu Gulf University, Qinzhou, 535000 China; 2 School of Petroleum and Chemical Engineering, Beibu Gulf University, Qinzhou, 535000 China; 3 School of Economics, Shanghai University, Shanghai, 201900 China

**Keywords:** Urban green transition, Production-side, Consumption-side, Agglomeration-side, Digital economy, Artificial intelligence, Economics, Economics, Environmental social sciences, Geography, Geography

## Abstract

This study examines the pivotal role of artificial intelligence (AI) in advancing the urban green transition (UGT), with a particular focus on the Yangtze River Delta (YRD) Urban Agglomeration. Drawing on panel data from 40 cities in the YRD between 2012 and 2024, the research utilizes benchmark regression, non-linear effect analysis, and spatial econometric models to investigate how AI influences UGT through production, consumption, and agglomeration channels. The main findings are as follows: (1) AI exerts a significant positive effect on UGT. (2) UGT exhibits strong spatial autocorrelation, whereas AI’s impact is characterized by robust local promotion but limited spatial spillover. (3) The influence of AI displays marked heterogeneity, varying by urban hierarchy, geographic location, resource endowment, and levels of institutional and market development. (4) The effects of AI on UGT are mediated by fixed capital stock, industrial structure upgrading, and consumption levels. Additionally, digital infrastructure, green consumption awareness, and digital industry agglomeration serve as moderating factors. AI’s influence also exhibits nonlinear diminishing marginal effects at specific thresholds in productive services and agricultural agglomeration. These results highlight the multifaceted mechanisms through which AI drives sustainable urban development and offer policy implications for fostering green transformation in metropolitan regions.

## Introduction

 The intensification of global climate change, coupled with tightening resource and environmental constraints, constitutes a fundamental impediment to sustainable development. Historically, industrialization has followed a high-carbon, resource-intensive trajectory, which is increasingly incompatible with global carbon-neutrality imperatives^1^. In this context, the Urban Green Transition (UGT) has evolved from a localized environmental strategy into a systemic socio-economic paradigm shift, requiring the decoupling of economic growth from environmental degradation^2^.In response to these severe ecological pressures, the Chinese government—as evidenced by the Opinions of the State Council on Accelerating the Comprehensive Green Transition of Economic and Social Development—has prioritized the construction of climate-adaptive cities and the enhancement of urban-rural resilience, explicitly promoting green construction methods and comprehensive pollution governance.

Within this context, artificial intelligence (AI) has emerged as a transformative technological force. AI reshapes traditional production functions through autonomous learning, precise algorithms, and massive-scale data processing^3^. By optimizing energy allocation and significantly improving production efficiency, AI offers a direct pathway to mitigate pollution emissions. However, the research motivation of this study stems from an unresolved paradox: while AI improves operational efficiency, its high energy demand and “rebound effect” may inadvertently exacerbate carbon footprints^4^. Thus, investigating whether AI can function as a novel engine to drive UGT holds profound contemporary value for achieving high‑standard economic progress.

Existing scholarship has extensively explored the drivers of UGT, primarily focusing on factors including Information and Communication Technology (ICT), carbon abatement strategies, and institutional reforms^5,6^. However, while previous studies have examined the broad impacts of digitalization, the specific role of AI—as distinct from general ICT—in fostering green transformation within a regional network remains underexplored. A critical innovation of this paper is the introduction of the Urban Agglomeration perspective. We argue that UGT is not an isolated urban event but a systemic process shaped by spatial interdependencies and network externalities^7^.

The Yangtze River Delta (YRD) urban agglomerations are selected as the primary laboratory for this study for three reasons. First, as China’s most integrated UA, it possesses the highest density of AI industrial clusters, offering a rich data landscape for empirical testing. Second, the YRD urban agglomerations exhibit a significant “environmental gradient” from the developed coastal hubs to the transitioning inland peripheries, allowing for a robust heterogeneity analysis. Third, the YRD’s integrated regional strategy serves as a global benchmark for balancing rapid digitalization with strict environmental regulations. This research seeks to close the identified research gap by systematically examining the pathways through which AI impacts UGT. From a theoretical perspective, urban agglomeration provides an essential spatial carrier and network environment for AI to exert green empowerment effects on UGT. Relying on industrial agglomeration, factor flows, and spatial network externalities within urban agglomerations, AI breaks the geographical boundaries of green technology diffusion, realizes cross-city spillovers, and further promotes coordinated UGT across the entire region. In turn, the advancement of UGT optimizes the spatial layout and ecological governance level of urban agglomerations, forming a mutually interactive theoretical relationship among AI, UGT, and urban agglomeration. The theoretical significance lies in integrating AI as a distinct spatial variable into the New Economic Geography (NEG) and Directed Technical Change frameworks, while its practical value manifests in providing a policy toolkit for regional sustainable development.

## Literature review and research hypotheses

###  Literature review

Existing research on AI has largely focused on sectoral implications, such as the green transformation of manufacturing enterprises, urban green economic development, and energy‑environmental performance^8^. Scholars have also explored AI’s applications in urban observation and imaging, innovative land-use management, and broader urban sustainable development^9,10^. However, direct investigations of AI’s holistic impact on UGT remain scarce.In the broader UGT literature, various institutional and economic drivers have been identified. Regarding institutional factors, Li et al. found that media scrutiny and environmental regulations amplify the positive effects of mixed-ownership reform on total factor productivity under ecological constraints^11^. In terms of policy drivers, Liu et al. revealed that “zero‑waste city” pilot policies accelerate UGT by fostering green technological innovation^12^. From an economic geography perspective, Wu demonstrated that digitalization generates significant cross-boundary externalities that benefit UGT in neighboring cities^13^, while Fu et al. compared the differential impacts of education and technology on UGT across Southeast and Northeast China^13^. Methodologically, UGT research mainly focuses on performance evaluation and driver identification using spatial and econometric models. For performance evaluation, Wang et al. applied the entropy-weighted TOPSIS model to assess resource‑based cities^15^, and Li et al. gauged UGT levels by measuring the efficiency of urban green space utilization^14^. Xiong et al. used the Global Malmquist-Luenberger (GML) index to estimate urban green total factor productivity as a proxy for high-quality development^15^. Regarding driving mechanisms, scholars have employed diverse tools: Ding et al. used spatial structure analysis to investigate determinants of urban carbon emissions, Chen et al. applied the Shapley value decomposition method^18^, and Wang et al. examined government environmental audits using a System Gaussian Mixture Model^16^. Another study by Wang et al. applied Exploratory Spatial Data Analysis (ESDA) and the Spatial Durbin Model (SDM) to assess the impact of population aging^17^. Zhang et al. combined the DID method with mediation models to quantify the performance of low‑carbon development^18^. Despite these advances, two critical limitations remain. First, the nexus between AI and UGT is underexplored; existing discussions focus on specific sub‑sectors or general innovative city management, lacking a dedicated analysis of how AI specifically empowers comprehensive UGT. Second, methodologically, few studies systematically integrate spatial econometric models (SEM) to comprehensively evaluate the spatiotemporal evolution and underlying mechanisms of AI’s effects on UGT.

This study bridges these gaps by examining the pathways through which AI affects UGT and assessing the presence of significant spatial heterogeneity and spillover effects. Building a theoretical framework from the production-consumption-gglomeration perspective, we empirically investigate the intrinsic driving forces and spatial transmission mechanisms of AI. The study’s significance is twofold. Theoretically, it elucidates the micro-mechanisms by which AI empowers UGT, thereby filling a specific gap in understanding AI as a distinct technological factor driving urban sustainability. Empirically, it provides a holistic quantitative assessment of AI’s impact on UGT by synthesizing benchmark regression, spatial econometrics, and mechanism identification methods. This multi-dimensional approach overcomes the limitations of previous isolated methodologies, yielding a finer-grained understanding of UGT’s driving mechanisms.

###  Research hypotheses

#### The production-consumption-agglomeration framework

This study further clarifies the inherent theoretical logic among AI, UGT, and urban agglomeration. As a critical spatial carrier, the urban agglomeration shapes the boundary conditions for factor allocation, technology spillovers, and industrial collaborative development. As a revolutionary technological driver, AI reshapes production and consumption patterns and drives green innovation and energy-efficiency upgrades. Under the network externality and agglomeration-economy logic of New Economic Geography, urban agglomeration constrains and amplifies the green effect of AI, while AI-driven UGT further promotes the high-quality integrated development of urban agglomeration. The following hypotheses are proposed based on this theoretical nexus. To systematically evaluate the impact of AI on UGT, this study develops an integrated analytical framework encompassing production-side factors, consumption-side drivers, and agglomeration-side boundary conditions. Specifically, Fixed Capital Stock (FCS) and Industrial Structure Upgrading (ISU) are selected to represent supply-side evolution, while Consumption Level (CL) captures the demand-pull effect. Furthermore, Digital Infrastructure (DI), Green Consumer Awareness (GCA), and Market Heterogeneity are incorporated to define the socio-economic boundaries governing this transition.

#### Direct impact and spatial spillover effects

According to New Economic Geography (NEG), technological progress is not spatially neutral but precipitates significant localized externalities and cross-border spillovers. At the local level, AI effectively reduces information-matching frictions in factor markets, optimizes the allocation of capital, labor, and technological resources, and promotes the transformation of low-carbon production modes and the application of green innovation. By empowering industrial digitalization, optimizing energy utilization, and accelerating the penetration of green technology, AI can directly improve local UGT performance in the YRD. AI weakens geographical barriers to the diffusion of knowledge and technology, enabling green technology achievements and low-carbon management experiences originating from central cities to trickle down through industrial and spatial linkages, thereby continuously releasing local green development dividends^10^. AI diminishes the geographic friction of knowledge transfer, enabling green innovations from core hubs to generate a “trickle-down effect” across industrial chains^19^. Notably, the digitalization process exerts significant externalities across geographical boundaries, benefiting the UGT of neighboring cities^20^. From the spatial interaction perspective emphasized by New Economic Geography, digital evolution driven by AI possesses strong cross-boundary externality characteristics. It accelerates cross-regional flows of green knowledge, low-carbon technology, and other innovations, breaks down market segmentation among adjacent cities, and generates significant spatial spillover effects. Neighboring cities can share technological innovation achievements, optimize industrial collaborative layouts, and learn advanced green governance experiences through spatial correlation, thereby indirectly promoting their own UGT levels. Against this theoretical backdrop of spatial synergy and technological diffusion, the following hypothesis is formulated:

##### H1:

AI promotes UGT in the local city.

##### H2:

AI exerts a significant spatial spillover on UGT of geographically adjacent municipalities.

#### Mediating mechanisms

Based on the Directed Technical Change Theory, this study argues that AI reshapes the marginal productivity of various production factors, thereby triggering resource reallocation across production and consumption sectors, which further mediates the influence of AI on UGT^21^. From the supply-side perspective, AI-driven technological progress generates a prominent capital deepening effect and directly promotes the accumulation of regional fixed capital stock. The establishment and maintenance of digital infrastructure and intelligent equipment matched with fixed capital accumulation are inherently energy-intensive, which readily induces an energy rebound effect. Specifically, the scale expansion of fixed capital offsets the energy efficiency gains brought by AI technology, exacerbates energy consumption and carbon emissions, and ultimately inhibits urban green transformation, forming the intrinsic mediating logic of fixed capital stock. Meanwhile, AI advances the upgrading of industrial structure through technological iteration and factor reallocation, guiding the economic transition toward servitization and digitalization. Nevertheless, the structural transformation induced by AI may divert excessive capital, technology, and other factors into the virtual service sector, resulting in capital hollowing out within the manufacturing industry. As the core carrier of green renovation and carbon abatement, the manufacturing sector faces insufficient investment in green upgrading due to capital outflows, weakening the overall momentum of urban green transformation. Accordingly, AI exerts an indirect inhibitory effect on UGT through the upgrading of industrial configuration. Conversely, on the consumption side, by mitigating information asymmetry in the labor market and empowering labor skill upgrading, AI effectively narrows the income gap and optimizes income distribution. In line with the Directed Technical Change Theory, improvements in residents’ income levels and the optimization of the distribution structure drive the upgrading of consumption demand, shifting residents’ consumption preferences from basic material needs toward high-quality, low-carbon environmental amenities. The escalation of green consumption demand further creates a market-driven incentive mechanism, forcing companies to increase spending on green innovation and adopt cleaner production methods. Consequently, AI can indirectly facilitate UGT by elevating residents’ consumption levels^22^. Building upon this rationale of resource reallocation and structural contradictions, we propose:

##### H3a:

AI inhibits UGT by promoting fixed capital stock accumulation.

##### H3b:

AI inhibits UGT by driving industrial structure upgrading.

##### H3c:

AI promotes UGT by elevating resident consumption levels.

#### The moderating mechanisms

The efficacy of AI-driven UGT depends on institutional and market boundaries. Digital infrastructure serves as the physical platform; when optimized by AI algorithms, it evolves from an energy-intensive facility into an efficient carrier of green innovation^23^. As the fundamental physical carrier for AI applications and empowerment, digital infrastructure (DI) is typically energy-intensive, requiring substantial energy input and resource investment during initial construction. Due to inefficient allocation of digital resources and energy in the early stages, DI alone may adversely affect UGT. From the perspective of the Resource Complementarity Theory, AI technology and digital infrastructure exhibit strong complementary attributes. AI’s intelligent scheduling and optimization capabilities can be deeply integrated into DI operations, enabling efficient allocation of digital resources, reducing idle resource consumption and energy waste, and offsetting the direct negative effects of DI. This complementary coupling enhances DI’s role in supporting AI’s green empowerment, strengthens AI’s marginal contribution to UGT, and thereby generates a beneficial moderating role on the AI-UGT relationship. Similarly, green consumption awareness (GCA) functions as a complementary soft resource for AI’s green application. GCA shapes market environmental preferences and innovation incentive structures, and its alignment with AI’s technological advantages forms a market-oriented complementary mechanism. Specifically, GCA guides enterprises to direct AI’s computational and innovative capabilities toward green product research and development, low-carbon production, and full-lifecycle carbon management, while AI provides technical support to help enterprises meet market green demand. This synergy strengthens market screening of AI green applications, boosts enterprise motivation to adopt AI for low-carbon transformation, and thus reinforces AI’s promotional effect on UGT. Regarding digital industry agglomeration (DIA), its moderating effect also follows the logic of resource complementarity. Moderate DIA aggregates digital resources, technical talent, and AI application scenarios, forming a complementary resource ecosystem that reduces the cost of popularizing and applying AI and facilitates mutual complementarity between AI and digital industry resources, thereby supporting AI’s green empowerment. Excessive DIA, however, leads to an over-concentration of regional digital resources, triggering fierce competition for talent, capital, and environmental carrying capacity, resulting in a mismatch and waste of complementary resources. Simultaneously, excessive agglomeration squeezes resource inputs for the green upgrading of traditional industries, hindering effective complementary matching between AI technology and the UGT needs of these industries, and thus weakening the efficiency of AI’s green empowerment, thereby exerting a negative moderating effect on the AI-UGT nexus.

Consequently, derived from the aforementioned theoretical discussion from the perspective of Resource Complementarity Theory, we formulate the following research hypotheses, which are fully consistent with the subsequent moderating effect test results:

##### H4a:

Digital infrastructure (DI) exerts a positive moderating influence on the impact of AI on UGT.

##### H4b:

Green consumption awareness (GCA) serves as a positive moderator for the influence of AI on UGT.

##### H4c:

Excessive digital industry agglomeration (DIA) has a detrimental moderating impact upon AI on UGT.

#### Non-linear threshold logic: from individual substitution to systemic synergy

The green empowerment effect of AI on UGT is heterogeneous, constrained by significant threshold effects at different levels of industrial agglomeration. According to Directed Technical Change Theory, the green technological return of AI is highly sensitive to the scale of industrial agglomeration and factor-matching efficiency^24^. At the outset of industrial co‑location, industrial distribution is relatively scattered, and factor allocation appears fragmented, resulting in an insufficient supply of AI application scenarios and high marginal costs when AI is embedded into production and operation processes. At this stage, AI only operates on partial links, such as simple mechanical substitution and single-factor efficiency improvements. Its green empowerment is mainly reflected in the direct driving effect, generating a strong marginal contribution to UGT. Once the industrial agglomeration level crosses the critical threshold, the evolutionary logic of AI’s green empowerment shifts from individual substitution to systemic synergy, which is realized through Jacobs externalities and scale economies emphasized by New Economic Geography^25^. On the one hand, industrial agglomeration stimulates the concentration of high-end factors, including talent, technology, and data, forms a complete industrial chain and factor matching ecosystem, effectively reduces information friction and transaction costs in AI applications, and enables AI to deeply penetrate the whole process of green production, green innovation, and green governance. On the other hand, knowledge spillovers and labor pooling effects brought about by agglomeration accelerate the iterative upgrading of AI green technology, promote the collaborative application of AI across enterprises, and further amplify the effectiveness of AI green empowerment. In the context of agricultural agglomeration, AI is primarily applied to specialized scenarios such as precision irrigation and pest control, exerting a strong positive driving effect on UGT. When agricultural agglomeration crosses the threshold, although the scale effect of agglomeration further expands AI application scenarios, the excessive expansion of agricultural scale aggravates environmental pressure, including agricultural non‑point source pollution, and weakens the marginal empowerment effect of AI. Based on the evolutionary logic that AI transforms from individual substitution below the threshold to systemic synergy above the threshold, as well as the heterogeneous threshold characteristics of the two types of industrial agglomeration, the following research hypotheses are proposed:

##### H5:

AI exerts a non-linear effect on UGT, with increasing marginal returns, conditional on levels of agricultural and producer service agglomeration.

#### Market heterogeneity theoretical analysis

The market environment is jointly shaped by market potential, market integration, and market segmentation. According to Factor Allocation Theory, the market environment fundamentally determines the efficiency of urban factor allocation and the incentive structure for corporate technological innovation, thereby constraining and moderating the effectiveness of AI in promoting green transition. Differences in factor allocation efficiency directly determine whether AI technology can be effectively implemented and fully utilized, ultimately resulting in divergent impacts of AI on UGT. The specific mechanisms are elaborated as follows.

High market potential implies a larger scale of demand, stronger economic agglomeration, and more sufficient factor supply. From the perspective of Factor Allocation Theory, adequate factor supply enables efficient factor matching and generates economies of scale, thereby reducing the costs of promoting and applying AI technology. This provides sufficient motivation and resource support for enterprises to adopt AI for green production upgrading, facilitates the efficient combination of AI with green production factors, and thus delivers a robust positive motivational effect on green transition. In contrast, cities with medium or low market potential are constrained by limited demand scale, insufficient factor agglomeration, and weak technological absorptive capacity, resulting in low allocative efficiency. Although AI may still exert a positive influence, the lack of supporting conditions, such as capital, talent, and application scenarios, hinders the effective allocation of AI and green factors, making it difficult to generate a stable and significant driving effect.

##### H6:

The positive impact of AI on UGT is strongest in cities with high market potential.

High market integration reduces inter‑regional transaction costs, breaks down market barriers, and promotes the diffusion of AI technology, green production factors, and advanced management experience. According to Factor Allocation Theory, a higher degree of market integration enables smoother cross‑regional factor flows, achieves the cross‑regional optimal allocation of AI technology and green factors, allows cities to fully absorb external technology spillovers, improves factor allocation efficiency, and thereby strengthens the green-enabling effect of AI. Regions with medium or low integration, however, are constrained by local protectionism and factor market segmentation, which impede factor mobility and reduce allocation efficiency, thereby hindering the free flow and effective allocation of AI technology and green factors.

##### H7:

 The favorable effect of AI on UGT is strongest in regions with high market integration.

High market segmentation tends to create local monopolies, which, to some extent, exclude external market competition and force local enterprises to rely on technological innovation to overcome development bottlenecks. Under a local monopoly, local factors cannot be optimally allocated through cross‑regional flows; thus, enterprises must focus on the efficient utilization of internal factors. As an efficient clean technology, AI helps enterprises optimize internal factor allocation, reduce production costs and environmental pollution, and enhance core competitiveness, thereby significantly advancing the green transition. By comparison, in regions with moderate or low segmentation, market competition is relatively weak, and enterprises can compensate for internal deficiencies through external factor mobility. This reduces their incentive to adopt AI for internal resource optimization and green innovation. Although AI still shows a positive tendency, insufficient motivation and intensity of enterprise adoption weaken the optimization of factor allocation, and such effects often fail to pass significance tests.

##### H8:

The upward impact of AI on UGT is strongest in cities with high market segmentation.

**Fig. 1:**
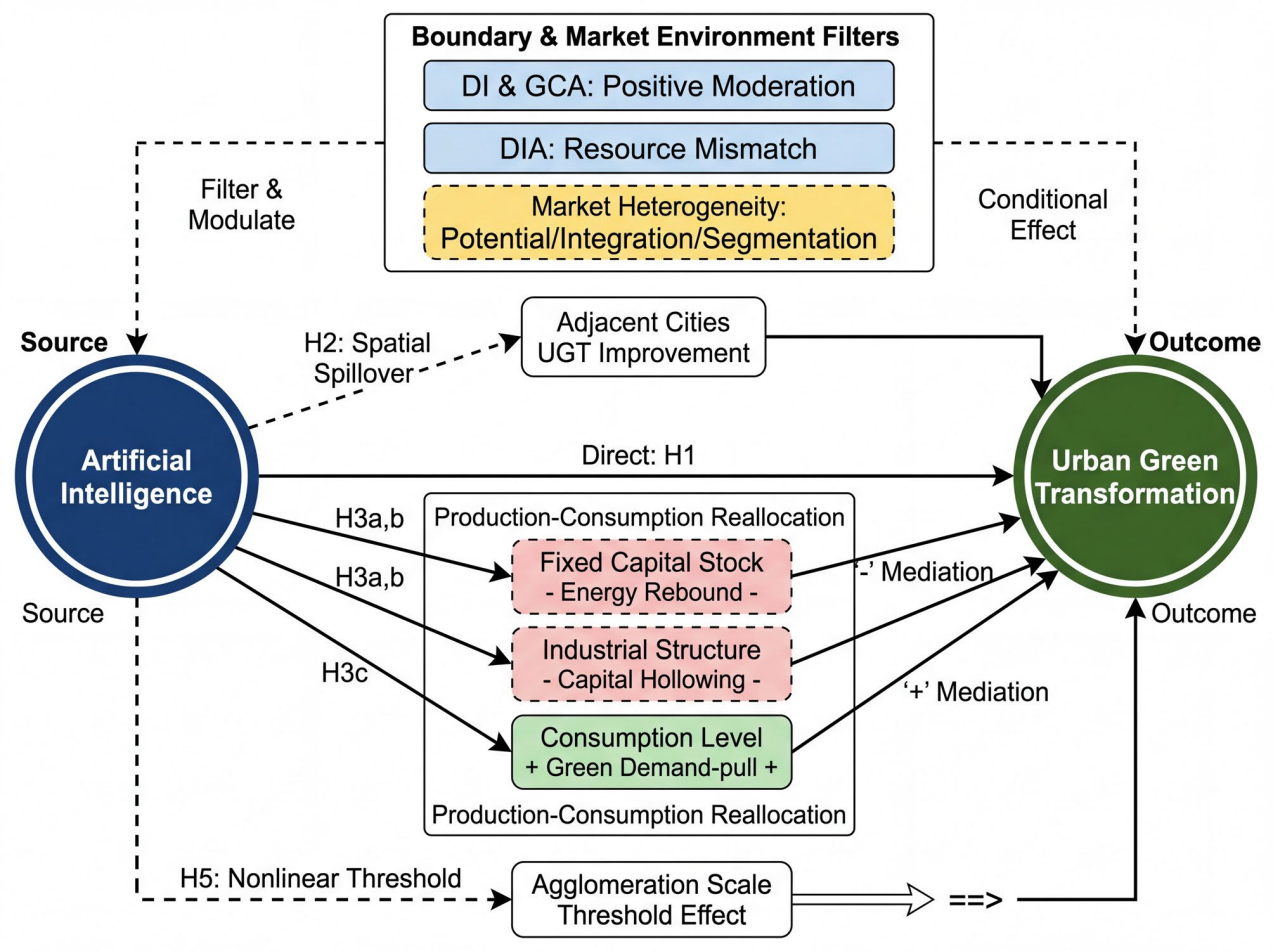
The theoretical framework. Solid lines denote direct/mediating effects, dashed lines denote moderating/heterogeneous effects, and double lines denote nonlinear threshold effects.

In summary, this paper develops a comprehensive theoretical framework that integrates six core mechanisms: direct effects, spatial spillovers, mediating pathways, moderating boundaries, nonlinear thresholds, and market heterogeneity. Grounded in Directed Technical Change Theory, New Economic Geography, Resource Complementarity Theory, and Factor Allocation Theory, the framework systematically explains the selection of mediating, moderating, threshold, and heterogeneous variables from the production, consumption, and agglomeration dimensions. Specifically, AI influences urban green transition through three mediating channels—fixed capital stock accumulation, industrial structure upgrading, and resident consumption level improvement—while digital infrastructure, green consumption awareness, and digital industry agglomeration serve as boundary moderators. Furthermore, productive service and agricultural agglomeration impose nonlinear threshold constraints, and market heterogeneity is examined with respect to market potential, market integration, and market segmentation. The complete framework is illustrated in Fig. 1.

## Research methodology

### Model specification

#### TOPSIS

This analysis relies on the TOPSIS to quantify UGT. As a multi-criteria decision-making approach, TOPSIS can synthesize multiple conflicting indicators into a single, quantifiable composite index, making it highly suitable for evaluating the complex, multidimensional developmental levels of UGT. In contrast to the traditional entropy method, this approach utilizes geometric distance to quantify inter-urban disparities with greater precision and objectivity, thereby boosting the validity and descriptive utility of the evaluation results. The specific calculation formulas refer to the work of Foley^26^ and are omitted here for the sake of brevity.

#### Baseline regression model

To probe the direct incidence of AI on UGT within the YRD Urban Agglomeration, this study constructs the following econometric model:

1 $$\:{UGT}_{ijt}={\alpha\:}_{1}+{\beta\:}_{1}{AI}_{it}+\sum\:_{k=1}^{n}{\theta\:}_{1k}{X}_{ikt}+{\mu\:}_{1i}+{\delta\:}_{1t}+{\epsilon\:}_{1it}$$

In Eq. (1), $\:{UGT}_{ijt}$ signifies the level of UGT of city i in dimension j at time t; $\:{AI}_{it}$ represents the AI level of city i at time t; $\:{X}_{ikt}$ signifies the k-th control variable for city i at time t; $\:{\epsilon\:}_{1it}$is the random disturbance term varying across individuals and time; $\:{\alpha\:}_{1}\:$is the intercept term; $\:{\beta\:}_{1}$and $\:{\theta\:}_{1k}$ are the coefficients of interest.

#### Spatial econometric model

The contribution of AI to UGT is characterized not only by direct effects but also by its potential to inform a spatial econometric model. Consequently, this research utilizes Moran’s I to assess the spatial dependence of UGT within the YRD Urban Agglomeration. The specific functional form is:

2 $$\:\mathrm{Moran's\:}I=\frac{n}{\sum\:_{i=1}^{n}\:\sum\:_{j=1}^{n}\:{w}_{ij}}\cdot\:\frac{\sum\:_{i=1}^{n}\:\sum\:_{j=1}^{n}\:{w}_{ij}\left(UG{T}_{i}-\overline{UGT}\right)\left(UG{T}_{j}-\overline{UGT}\right)}{\sum\:_{i=1}^{n}\:{\left(UG{T}_{i}-\overline{UGT}\right)}^{2}}$$

Where *\:n* is the number of cities, and$\:\:{w}_{1ij}$ represents the spatial weight matrix, and $\:\overline{UGT}$ is the sample mean. This study employs three distinct matrices to ensure robustness: the Economic Distance Matrix (EDM), the Economic Weight Matrix (EWM), and the Economic and Geographic Nested Matrix (EGNM). The specific formulas for these three matrices are presented as follows:

The Economic Distance Matrix (EDM) combines geographic distance and economic distance. where $\:{d}_{ij}$ is the geographic distance (based on longitude and latitude) and $\:GD{P}_{i}$ denotes the average per capita GDP of city *\:i*. The specific formulation is presented in Eq. (3):

3 $$\:{W}_{ij}^{\mathrm{econ}}=\left\{\begin{array}{c}\frac{1}{{d}_{ij}^{2}}\cdot\:\frac{1}{\left|{\mathrm{G}\mathrm{D}\mathrm{P}}_{i}-{\mathrm{G}\mathrm{D}\mathrm{P}}_{j}\right|}\hspace{0.25em}\hspace{0.25em}\hspace{0.25em}\hspace{0.25em}(i\ne\:j)\\\:0\hspace{0.25em}\hspace{0.25em}\hspace{0.25em}\hspace{0.25em}(i=j)\end{array}\right.$$

The Economic Weight Matrix (EWM) quantifies the relative importance of the combined economic magnitudes of cities *\:i* and *\:j* within the entire region. Where $\:{E}_{i}\:$and$\:\:{E}_{j}$ denote the per capita GDP of city *\:i* and city *\:j* over the period 2012–2024, and N represents the total number of cities; and $\:\sum\:_{k=1}^{N}\:{E}_{k}$ corresponds to the aggregate sum of economic indicators across all cities. The specific formulation is presented in Eq. (4):

4 $$\:{\mathbf{W}}_{ij}^{\mathrm{E}\mathrm{W}\mathrm{M}}=\left\{\begin{array}{c}\frac{{E}_{i}+{E}_{j}}{\sum\:_{k=1}^{N}\:{E}_{k}}\hspace{0.25em}\hspace{0.25em}\hspace{0.25em}\hspace{0.25em}(i\ne\:j)\\\:0\hspace{0.25em}\hspace{0.25em}\hspace{0.25em}\hspace{0.25em}(i=j)\end{array}\right.$$

The Economic and Geographic Nested Matrix (EGNM) simultaneously captures both geographical proximity and the intensity of economic linkages. Specifically, $\:{\stackrel{-}{GDP}}_{i}$ denotes the average GDP per capita of city *\:i* over the period 2012–2024, and $\:\sum\:_{k=1}^{N}\:{\stackrel{-}{GDP}}_{k}$represents the overall average GDP per capita across all cities. In this context, k refers to the distance decay coefficient in $\:\frac{1}{{d}_{ij}^{k}}\:$while typically taking the value of k=1or k = 2, this study adopts k = 2. N represents the total number of cities. The specific formulation is presented in Eq. (5):

5 $$\:{W}_{ij}^{\mathrm{nested}}=\left\{\begin{array}{c}\frac{1}{{d}_{ij}^{k}}\cdot\:\frac{{\stackrel{-}{GDP}}_{i}}{\sum\:_{k=1}^{N}\:{\stackrel{-}{GDP}}_{k}}\hspace{0.25em}\hspace{0.25em}\hspace{0.25em}\hspace{0.25em}(i\ne\:j)\\\:0\hspace{0.25em}\hspace{0.25em}\hspace{0.25em}\hspace{0.25em}(i=j)\end{array}\right.$$

To investigate the spatial spillover effects of AI on UGT, the Spatial Durbin Model (SDM) is applied, which includes spatially lagged terms for both the response and predictor variables. The SDM is specified as presented in Eq. (6):

6 $$\begin{gathered} UGT_{{it}} = \rho \mathop \sum \limits_{{j = 1}}^{N} w_{{ij}} UGT_{{jt}} + \beta _{1} AI_{{it}} + \mathop \sum \limits_{{k = 1}}^{n} \theta _{k} X_{{ikt}} + \mathop \sum \limits_{{j = 1}}^{N} w_{{ij}} \left( {\gamma _{1} AI_{{jt}} + \mathop \sum \limits_{{k = 1}}^{n} \gamma _{k} X_{{jkt}} } \right) + \mu _{i} + \delta _{t} + \left( { \in _{t} } \right) \hfill \\ \varepsilon _{{it}} = \lambda \mathop \sum \limits_{{j = 1}}^{N} w_{{ij}} \varepsilon _{{jt}} + \nu _{{it}} ,\nu _{{it}} \sim N\left( {0,\sigma ^{2} I_{n} } \right) \hfill \\ \end{gathered}$$

In Eq. (6), where$\:\:\rho\:\:$denotes the spatial lag parameter of the response variable; $\:{w}_{ij}\:$ is the spatial weight matrix element; $\:{\gamma\:}_{1}\:$and $\:{\gamma\:}_{k}\:$are the estimated effects of spatially lagged predictors; $\:\lambda\:\:$captures the spatial autocorrelation of the error term; $\:{\mu\:}_{i}$ and $\:{\delta\:}_{t}\:$denote city and time fixed effects, $\:{\epsilon\:}_{it}$ and $\:{\nu\:}_{it}$ are error terms.

#### Mediation effect model

To test whether AI influences UGT through mediating channels (e.g., technological innovation, industrial structure upgrading, or energy efficiency improvement), we adopt the stepwise mediation approach developed by Cheong (2001)^27^. The model consists of three Eqs. (7)-(9):

7 $$\:UG{T}_{it}={c}_{1}A{I}_{it}+\sum\:_{k=1}^{n}\:{\alpha\:}_{k}{X}_{ikt}+{\mu\:}_{1i}+{\delta\:}_{1t}+{\epsilon\:}_{1it}$$

8 $$\:{M}_{it}={a}_{1}A{I}_{it}+\sum\:_{k=1}^{n}\:{\beta\:}_{k}{X}_{ikt}+{\mu\:}_{2i}+{\delta\:}_{2t}+{\epsilon\:}_{2it}$$

9 $$\:UG{T}_{it}={c}_{1}^{{\prime\:}}A{I}_{it}+{b}_{1}{M}_{it}+\sum\:_{k=1}^{n}\:{\gamma\:}_{k}{X}_{ikt}+{\mu\:}_{3i}+{\delta\:}_{3t}+{\epsilon\:}_{3it}$$

Here, $\:{M}_{it}$ denotes the intermediate variable. The indirect effect is given by $\:{a}_{1}\times\:{b}_{1}$, and the direct effect by $\:{c}_{1}^{{\prime\:}}$. The significance of the indirect pathway is verified using the Sobel test or bootstrap confidence intervals.

####  Moderation effect model

To examine whether the impact of AI on UGT is contingent upon a moderating variable $\:{Z}_{it}$, we introduce an interaction term between AI and $\:{Z}_{it}$. The moderation effect model is specified as:

10 $$\:UG{T}_{it}={\alpha\:}_{2}+{\beta\:}_{2}A{I}_{it}+{\beta\:}_{3}{Z}_{it}+{\beta\:}_{4}\left(A{I}_{it}\times\:{Z}_{it}\right)+\sum\:_{k=1}^{n}\:{\delta\:}_{k}{X}_{ikt}+{\mu\:}_{4i}+{\delta\:}_{4t}+{\epsilon\:}_{4it}$$

In Eq. (10), the coefficient $\:{\beta\:}_{4}$ captures the moderating effect. A statistically significant $\:{\beta\:}_{4}$ indicates that the marginal effect of AI on UGT varies with the level of $\:{Z}_{it}$. In the empirical analysis, we also plot the marginal effects at different quantiles of the moderator to facilitate interpretation.

#### Threshold effect model

Following Bin^28^, we further explore potential non‑linear relationships by estimating a panel threshold model. The basic specification with a single threshold is:

11 $$\:UG{T}_{it}={\alpha\:}_{3}+{\phi\:}_{1}A{I}_{it}\cdot\:I\left({q}_{it}\le\:\gamma\:\right)+{\phi\:}_{2}A{I}_{it}\cdot\:I\left({q}_{it}>\gamma\:\right)+\sum\:^{n}\:{\psi\:}_{k}{X}_{ikt}+{\mu\:}_{5i}+{\delta\:}_{5t}+{\epsilon\:}_{5it}$$

In the equations (11), where $\:{q}_{it}$ is the threshold variable; $\:\gamma\:$ is the unknown threshold parameter; $\:I(\cdot\:)$ is an indicator function that equals 1 when the condition holds and 0 otherwise; $\:{\phi\:}_{1}\:$and $\:{\phi\:}_{2}\:$capture the effects of AI on UGT below and above the threshold, respectively. Multiple thresholds can be specified by adding additional regime‑switching terms. The threshold value $\:\gamma\:$ is estimated by minimizing the residual sum of squares, and its significance is tested using a bootstrap procedure.

### Variable description

#### Dependent variable

Existing studies on the measurement of UGT mainly focus on the production front, living, society, and the ecological environment. Wen et al. constructed an evaluation framework from the perspective of production–living–ecological space, but neglected to account for carbon emissions from industrial production and residential daily activities^29^. Mu et al. established an indicator system for urban economic green transformation following the logic of economic–social–environmental coordinated development; nevertheless, their framework failed to incorporate the substantial influence of energy consumption on socioeconomic and ecological performance^30^. Han et al. assessed UGT from the eco-efficiency total factor productivity perspective, while ignoring the inherent trade-off whereby coal consumption boosts economic growth but aggravates carbon emission levels. In addition, the indicator system developed by Mu et al. focused solely on the integrated development of UGT, without sufficiently capturing the actual impact of energy consumption on carbon emission abatement^31^. On the basis of the above literature, this paper further incorporates indicators related to total energy consumption and coal consumption. Guided by the goal of urban sustainability, a comprehensive UGT evaluation system is established that covers five dimensions: pollution reduction, carbon abatement, greening enhancement, green living, and urban transformation quality. Referring to Zheng et al., industrial SO₂ emissions, industrial NOₓ emissions, ammonia nitrogen emissions in wastewater, comprehensive utilization rate of general industrial solid waste, harmless treatment rate of municipal solid waste (MSW), and CO₂ emissions are selected to reflect the level of pollution and carbon emission reduction^32^. Following Shi et al., the per capita afforestation area, per capita park green space, green coverage rate in built-up areas, ratio of R&D expenditure to general fiscal budget revenue, per capita disposable income of residents, and tertiary industry added value are used to characterize the level of urban green development. In line with Hu et al., the economic output intensity of construction land is measured by the proportion of construction land area to GDP, and the per head built‑up zone is employed to reflect urban transformation quality^33^. According to Li et al., per capita built-up area and per capita road area are used to denote the level of green transition and green living, respectively^34^. Furthermore, the proportion of tertiary industry added value in GDP is chosen to represent industrial structure upgrading; the share of high-tech industry output value in GDP is applied to reflect the scale of high-tech industry development; and the proportion of employment in the tertiary industry to total employment is adopted to capture the employment structure of the tertiary industry. All these indicators jointly constitute the evaluation dimension of urban green transformation quality. The precise metric specifications and classifications are presented in Table 1.

**Table 1:** Evaluation indicator system for UGT.

Level 1 Indicators	Level 2 Indicators	Level 3 Indicators	Direction	Unit	Weight
UGT	Pollution Reduction	Industrial SO_2_ emissions	-	Tons	0.003
		Industrial NO_X_ emissions	-	Tons	0.003
		Ammonia nitrogen emissions in wastewater	-	10^4^tons	0.005
		Comprehensive utilization rate of general industrial solid waste	+	%	0.007
		Harmless treatment rate of municipal solid waste (MSW)	+	%	0.001
	Carbon Reduction	CO2 emissions/GDP	-	10^4^ tons /10^8^ CNY	0.007
		CO2 emissions/ administrative area	-	10^4^ tons /Km^2^	0.005
		CO2emissions / Total population	-	10^4^ tons /10^4^ persons	0.005
		Total energy consumption /GDP	-	10^4^ tce /10^8^ CNY	0.005
		Total coal consumption /GDP	-	10^4^ tce /10^8^ CNY	0.003
		Ratio of total energy consumption to coal consumption	-	10^4^ tce /10^4^ tons	0.002
		Per capita electricity consumption	-	kWh / person	0.006
		Per capita water consumption	-	M^3^/ person	0.017
	Greening Enhancement	Per capita afforestation area	+	Hectare/person	0.220
		Per capita park green space	+	Hectare/person	0.100
		Green coverage rate in built-up areas	+	%	0.015
		Ratio of R&D expenditure to general budget revenue	+	%	0.051
		Per capita disposable income of residents	+	CNY	0.053
		Value added of the tertiary industry /GDP	+	10^8^ CNY	0.034
	Green Living	Gas penetration rate	+	%	0.002
		Water penetration rate	+	%	0.001
		Per capita road area	-	M^2^/ person	0.014
	Urban Transformation Quality	Industrial structure upgrading	+	%	0.040
		Employment share of the tertiary industry	+	%	0.033
		Output share of high-tech industry	+	%	0.199
		Economic output intensity of construction land	+	10⁸CNY/km²	0.037
		Per capita built-up area	-	m²/person	0.001
		Number of invention patents per 10,000 population	+	piece	0.134

Note: “+” for positive contribution to UGT, “–” for negative contribution to UGT.

#### Explanatory variable

AI development is a multidimensional and systematic undertaking—factors such as input, talent support, industrial applications, and innovative outputs. Most existing studies use a single proxy indicator to measure the level of AI development, which fails to capture its comprehensive nature. For example, some studies use the number of AI enterprises as a standalone measurement dimension, ignoring the supporting roles of infrastructure, technological investment, and human capital^35^. Other scholars separately employ AI patent applications or industrial robot density to proxy AI development; however, such single-dimensional indicators cannot fully capture the overall input–output characteristics and systemic attributes of regional AI progress^36^. To address the limitations of single-proxy indicators and improve the rationality and representativeness of variable measurement, this study builds a multifaceted, integrated assessment framework for AI advancement, adhering to systematic criteria, representativeness, and data availability. Referring to mainstream analytical frameworks for the digital economy and intelligent transformation, we divide the AI evaluation system into input and output dimensions. The input dimension comprises AI development environment and AI talent resources, which characterize infrastructure conditions, fiscal science and technology investment, and human capital endowment for AI development. The output dimension includes industrial applications and innovative achievements, which objectively characterize the practical application effectiveness and technological innovation performance of AI. On this basis, corresponding tertiary indicators are selected, and the entropy weight method is applied to compute the comprehensive AI development index. This approach effectively avoids the subjective bias of subjective weighting and enhances the objectivity and robustness of variable measurement. Following Hu et al., Zhang et al., and Li et al.^37–39^, internet penetration rate, mobile phone penetration rate, and optical fiber cable density are adopted as proxy variables for digital infrastructure, which serves as the fundamental foundation for AI algorithm deployment and data transmission. Consistent with Sun et al.^40^, the number of AI enterprises and the installation density of industrial robots are used to reflect AI maturity and industrialization development. Following Hu and Feng et al.^37,41^, the workforce in information transmission, software, and IT services, along with the number of AI patents, are selected to characterize digital industry development and AI innovation capability, respectively. In line with Sun et al.^40^, local fiscal science and technology funding as a percentage of GDP and the number of higher education students per 100,000 population are adopted to measure regional innovation-driven development capacity. The complete hierarchical indicator system, specific measurement criteria, units, and corresponding weight results are presented in Table 2.

**Table 2:** Evaluation indicator system for AI.

Level 1 Indicators	Level 2 Indicators	Level 3 Indicators	Measurement Indicators	Unit	Weight
AI	Input	AI Development Environment	local fiscal science and technology expenditure/GDP	%	0.040
			Internet penetration rate	%	0.042
			Optical fiber cable density	km/km²	0.094
			Mobile phone penetration rate	%	0.034
			Number of AI enterprises	Count	0.241
		AI Talent Resources	Number of higher education students per 100,000 population	Person	0.050
			Employment in information transmission, software, and IT services	10^4^ persons	0.256
	Output	AI Industrial Application	Installation density of industrial robots	per 10,000 persons	0.021
		AI Innovation Achievements	Number of AI patents	Count	0.222

Note: The weight of each indicator is calculated by the entropy weight method.

#### Mediating variables

Given the multi-dimensional and complex impact of AI on the economy and society, this study selects Fixed Capital Stock (FCS), Industrial Structure Upgrading (ISU), and Consumption Level (CL) as mediators to investigate how AI affects UGT in the YRD urban agglomeration. Following Chen’s research methodology, FCS is computed as the natural logarithm of the regional fixed capital stock^42^. Following the perpetual inventory method (Bayer, 2006)^43^, this article calculates the capital stock using 2012 as the base year and investment deflators derived from the provincial price indices. This approach ensures comparability with previous studies. ISU is measured as the ratio of the tertiary industry’s added value to the secondary industry’s added value. A higher ratio indicates a more service‑oriented and advanced industrial structure. CL is defined as the ratio of the per capita disposable income of urban residents to that of rural residents. Although this indicator is often interpreted as a measure of consumption disparities, a smaller gap implies more balanced consumption capacity across urban and rural areas, which may facilitate a broader societal consensus on green transition. All mediating measures are extracted from the China City Statistical Yearbook and provincial statistical yearbooks over the period 2012–2024.

#### Moderating variables

To explore how AI realizes its potential for emission reduction and efficiency improvement, and based on the theoretical framework discussed above, this study selects Digital Infrastructure (DI), the Green Consumption Awareness (GCA), and Digital Industry Agglomeration (DIA) as the moderating variables. DI is evaluated from both input and output perspectives. Following the entropy method, we construct a composite index based on telecommunication business revenue, mobile phone penetration rate, and per capita internet broadband access ports. Higher DI values indicate better support for AI deployment from digital infrastructure. Following the approach of Yu et al.^44^, GCA is measured by the frequency of green‑concern keywords. This proxy captures the local government’s policy orientation towards green development. The calculation process for DIA is shown in Eq. (7), where the$\:{\mathrm{employed}}_{n}$ represent the number of digital employees in each city; $\:\sum\:{\mathrm{employed}}_{n}$ is the total number of digital employees in the region; $\:\sum\:\sum\:{\mathrm{employ}}_{n}$ is the total number of digital employees nationwide. INA is measured by the ratio of employment in the secondary industry to the administrative land area. The high-density agglomeration of producer services constructs a spatial network for knowledge spillovers. PSA is measured as a function of the number of employees in the tertiary industry, using the same method as in Eq. (12).

12 $$~agglomeration~_{n} = \frac{{~employed~_{n} /\sum ~employed~_{n} }}{{\sum ~employed~_{n} /\sum \sum ~employ~_{n} }}$$

#### Threshold variables

To investigate the non-linear characteristics of AI’s impact on UGT, this study, based on the theoretical framework discussed above, selects Productive Service Agglomeration (PSA) and Agricultural Agglomeration (AGA) as the threshold variables. Following the methodology of Chen^45^, Productive Service Agglomeration relies on Eq. (12) to compute the service-sector employee count; similarly, Agricultural Agglomeration is calculated using the number of employees in the primary industry, based on Eq. (12).

#### Control variables

Drawing on established research frameworks^46–48^, this research includes the following control variables: Population density, calculated as the ratio of the total population to the administrative area; Urban economic density, assessed as the ratio of GDP to the administrative area; Degree of openness, expressed as the ratio of total import and export volume to GDP; Human capital, represented by the proportion of students enrolled in regular institutions of higher education to the total population; and Healthcare level, proxied by the number of hospital beds per 100 people^49^. All control variables are sourced from the China City Statistical Yearbook and provincial yearbooks for the period 2012–2024.

###  Data sources

In accordance with the principles of research validity, reliability and data availability, this paper selects 40 cities in the YRD urban agglomeration as research samples, with the study period spanning from 2012 to 2024. Haozhou City is excluded from the sample due to severe missingness in core indicators. Macroeconomic and fundamental indicators, including local fiscal science and technology expenditure, GDP, the number of higher education students per 100,000 population, employment in information transmission, software, and information technology services, internet penetration rate, and mobile phone penetration rate, are mainly sourced from the China Statistical Yearbook and the China City Statistical Yearbook. Environmental indicators such as industrial SO_2_ emissions, industrial NO_X_ emissions, ammonia nitrogen emissions in wastewater, comprehensive utilization rate of general industrial solid waste, harmless treatment rate of municipal solid waste (MSW), and CO_2_ emissions are derived from the China Environmental Statistical Yearbook. Energy-related indicators, including total energy consumption, total coal consumption, the proportion of coal in total energy consumption, per capita electricity consumption, and per capita water consumption, are collected from the China Energy Statistical Yearbook. Land-use and ecological infrastructure parameters, such as construction land area, per capita built-up area, per capita afforestation area, per capita park green space area, green coverage rate in built-up areas, and optical fiber cable density, are obtained from the China Urban Construction Statistical Yearbook. Additional data are supplemented from the National Intellectual Property Administration of China (CNIPA), the EPS Database, and the CSMAR Database. Specific data sources for individual indicators are specified as follows: The word frequency of “digital economy” is extracted from municipal government work reports. The number of AI enterprises is compiled by retrieving AI-related keywords on the Tianyancha platform, retaining only enterprises with active or ongoing registration status. Industrial robot installation data, used to calculate robot installation density, are obtained from official reports provided by the International Federation of Robotics (IFR). For missing values of partial variables, linear interpolation is adopted for data imputation.

**Table 3:** Descriptive statistics.

Variable	Obs	Mean	Std. Dev.	Min	Max
UGT	520	0.939	0.100	0.065	0.996
AI	520	0.093	0.105	0.010	0.712
PD	520	0.127	0.118	0.007	0.717
UED	520	1.351	1.743	0.042	9.893
OD	520	0.268	0.365	0.005	2.887
HCL	520	1.992	1.566	0.227	9.533
MSL	520	0.504	0.174	0.209	1.183
GPA	520	1.224	1.330	-0.178	7.587
RFC	520	9.672	0.737	8.012	11.185
ISU	520	2.399	0.118	2.067	2.761
CL	520	0.245	0.243	0.013	1.884
GCA	520	8.743	0.180	7.630	9.626
DIA	520	0.594	0.660	0.082	4.181
AGA	520	0.000	0.000	0.000	0.001
INA	520	0.012	0.016	0.000	0.097
PSA	520	0.879	3.297	0.047	25.621

##  Research analysis

### Development patterns of UGT in the YRD urban agglomeration

In accordance with the State Council’s Notice on Adjusting the Classification Standards for City Sizes, the 40 cities within the YRD urban agglomeration are divided into four tiers: First-, Second-, Third-, and Fourth-tier cities. The specific results of this classification are presented in Table 4. Overall, the UGT level across the YRD urban agglomeration demonstrates significant imbalance and differentiation. Between 2012 and 2024, the UGT levels across 40 cities in the YRD underwent a profound evolution characterized by significant spatial non-uniformity. While overall green development improved, the persistent absolute gap between the highest and lowest UGT values highlights a deep-seated regional imbalance. During this period, internal rankings fluctuated sharply; while traditionally high-performing cities like Fuyang and Suzhou maintained relatively stable positions, others such as Huangshan faced severe transition bottlenecks, leading to a dramatic decline in their standing. A defining feature of this evolution is the “tier inversion” phenomenon, where third- and fourth-tier cities have consistently outperformed their first-tier counterparts. This trend is driven by the “late-mover advantage,” which allows lower-tier cities to rapidly enhance UGT through targeted industrial green-upgrading. Conversely, first-tier cities like Shanghai—despite their progress—face slower growth rates due to their highly service-oriented structures, immense urban scale, and the inherent complexity of transitioning legacy industrial systems. Ultimately, the YRD’s green transition landscape has been reshaped into a pattern in which lower-tier cities lead, and higher-tier cities face sustained pressure, a divergence rooted in varying industrial foundations and transition agility.

**Table 4:** Results of UGT in the YRD Urban Agglomeration.

2012				2024			
Category	City	UGT	Rank	Category	City	UGT	Rank
Third-tier cities	Fuyang	0.996	1	Fourth-tier cities	Suzhou	0.992	1
Fourth-tier cities	Suzhou	0.995	2	Fourth-tier cities	Maanshan	0.992	2
Third-tier cities	Huaibei	0.995	3	Third-tier cities	Fuyang	0.990	3
Third-tier cities	Huainan	0.991	4	Second-tier cities	Wuhu	0.986	4
Third-tier cities	Liuan	0.991	5	Third-tier cities	Xuancheng	0.984	5
Third-tier cities	Huangshan	0.991	6	Third-tier cities	Chizhou	0.980	6
Fourth-tier cities	Bengbu	0.984	7	First-tier cities	Shanghai	0.978	7
Third-tier cities	Xuancheng	0.981	8	Third-tier cities	Lishui	0.975	8
Third-tier cities	Chizhou	0.980	9	First-tier cities	Suzhou	0.972	9
Fourth-tier cities	Chuzhou	0.979	10	First-tier cities	Zhoushan	0.972	10
Fourth-tier cities	Anqing	0.979	11	Third-tier cities	Hefei	0.971	11
Fourth-tier cities	Tongling	0.975	12	Third-tier cities	Hangzhou	0.971	12
Fourth-tier cities	Maanshan	0.971	13	Second-tier cities	Jiaxing	0.968	13
Second-tier cities	Wuhu	0.966	14	Fourth-tier cities	Anqing	0.966	14
Third-tier cities	Hefei	0.965	15	Third-tier cities	Huaibei	0.963	15
First-tier cities	Zhoushan	0.964	16	Second-tier cities	Nanjing	0.959	16
Third-tier cities	Lishui	0.963	17	Third-tier cities	Huzhou	0.957	17
Fourth-tier cities	Quzhou	0.961	18	Third-tier cities	Yangzhou	0.952	18
Third-tier cities	Huzhou	0.958	19	Second-tier cities	Wenzhou	0.952	19
Third-tier cities	Jinhua	0.958	20	Fourth-tier cities	Tongling	0.951	20
Second-tier cities	Jiaxing	0.945	21	Second-tier cities	Xuzhou	0.950	21
First-tier cities	Taizhou	0.939	22	Fourth-tier cities	Shaoxing	0.950	22
Fourth-tier cities	Shaoxing	0.937	23	Second-tier cities	Yancheng	0.947	23
Second-tier cities	Wenzhou	0.929	24	Third-tier cities	Ningbo	0.946	24
Third-tier cities	Ningbo	0.925	25	First-tier cities	Taizhou	0.935	25
Third-tier cities	Hangzhou	0.924	26	Third-tier cities	Huainan	0.935	26
Second-tier cities	Xuzhou	0.923	27	First-tier cities	Zhenjiang	0.935	27
Second-tier cities	Suqian	0.922	28	Second-tier cities	Wuxi	0.935	28
Third-tier cities	Lianyungang	0.919	29	Third-tier cities	Huaian	0.934	29
Third-tier cities	Huaian	0.915	30	Second-tier cities	Taizhou	0.931	30
Second-tier cities	Taizhou	0.915	31	Third-tier cities	Changzhou	0.930	31
First-tier cities	Zhenjiang	0.914	32	Fourth-tier cities	Chuzhou	0.928	32
Third-tier cities	Yangzhou	0.912	33	Second-tier cities	Nantong	0.928	33
Second-tier cities	Yancheng	0.911	34	Third-tier cities	Liuan	0.926	34
Second-tier cities	Nantong	0.910	35	Fourth-tier cities	Quzhou	0.925	35
First-tier cities	Suzhou	0.890	36	Third-tier cities	Lianyungang	0.925	36
Third-tier cities	Changzhou	0.877	37	Second-tier cities	Suqian	0.891	37
Second-tier cities	Wuxi	0.873	38	Fourth-tier cities	Bengbu	0.882	38
Second-tier cities	Nanjing	0.836	39	Third-tier cities	Jinhua	0.872	39
First-tier cities	Shanghai	0.521	40	Third-tier cities	Huangshan	0.806	40

### Benchmark regression analysis

The empirical results demonstrate that AI contributes significantly to UGT, serving as a pivotal driver of regional sustainable development. In the baseline two-way fixed-effects model (1), the coefficient on AI is significantly positive, thereby validating Hypothesis H1. To ensure the reliability of these findings, several robustness checks were conducted. Model (2) incorporates the quadratic terms of control variables to examine potential non-linear effects, while Model (3) omits time fixed effects to test the stability of the core independent variable. In both specifications, the AI coefficients remain consistently and significantly positive. Furthermore, the models exhibit adequate explanatory power, with R^2^ values ranging from 0.160 to 0.197, confirming that the selected variables effectively capture the dynamic characteristics of urban green transition.

**Table 5:** Benchmark regression results.

Variable	UGT	UGT	UGT
	(1)	(2)	(3)
AI	0.538^***^	0.633^***^	0.437^***^
	(2.460)	(2.570)	(2.330)
Controls (Linear terms)	Yes	No	Yes
Controls (Quadratic terms)	No	Yes	No
Time Fixed Effects	Yes	Yes	No
Individual Fixed Effects	Yes	Yes	Yes
Cons	0.985^***^	0.940^***^	1.033^***^
	(17.020)	(42.000)	(21.490)
R^2^	0.169	0.170	0.137
rho	0.194	0.197	0.160
N	520	520	520

Note: t-statistics in parentheses. ^*^ *p* < 0.05, ^**^ *p* < 0.01, ^***^ *p* < 0.001.

### Spatial effects

####  Moran’s I

The analysis of the global Moran’s I indicates that the UGT of the YRD urban agglomeration exhibits a statistically significant positive spatial autocorrelation. This index rose steadily from 0.126 in 2012 to 0.251 in 2024, suggesting a progressively intensifying spatial agglomeration of green development within the region. Based on the evolution of LISA cluster maps, the spatial pattern has transitioned from early-stage dispersion to a highly structured morphology. In 2012, UGT exhibited a weak random distribution with minimal spatial spillover effects. By 2017, a contiguous High-High cluster belt emerged in southern Anhui, centered around Anqing and Chizhou, marking the initial formation of regional green growth poles; meanwhile, persistent Low-Low clusters in northern Jiangsu and southern Zhejiang highlighted a development “lock-in” in underdeveloped peripheral areas. By 2024, this spatial configuration will be further solidified, as the High-High cluster in southern Anhui expands into a continuous “Green Development Club,” demonstrating enhanced regional synergy. Conversely, the concentration of Low-Low clusters in northern Jiangsu and parts of southern Jiangsu reveals an unresolved structural divergence. Notably, the absence of significant High-Low or Low-High outliers throughout the study period suggests that the YRD’s transition is characterized by contiguous linkage rather than “isolated polarization.” In summary, the spatial pattern of UGT in the YRD has evolved into a highly aggregated and synergistic structure, yet the relative lock-in of high- and low-level clusters underscores the latent risk of a widening internal green development gap.

**Fig. 2:**
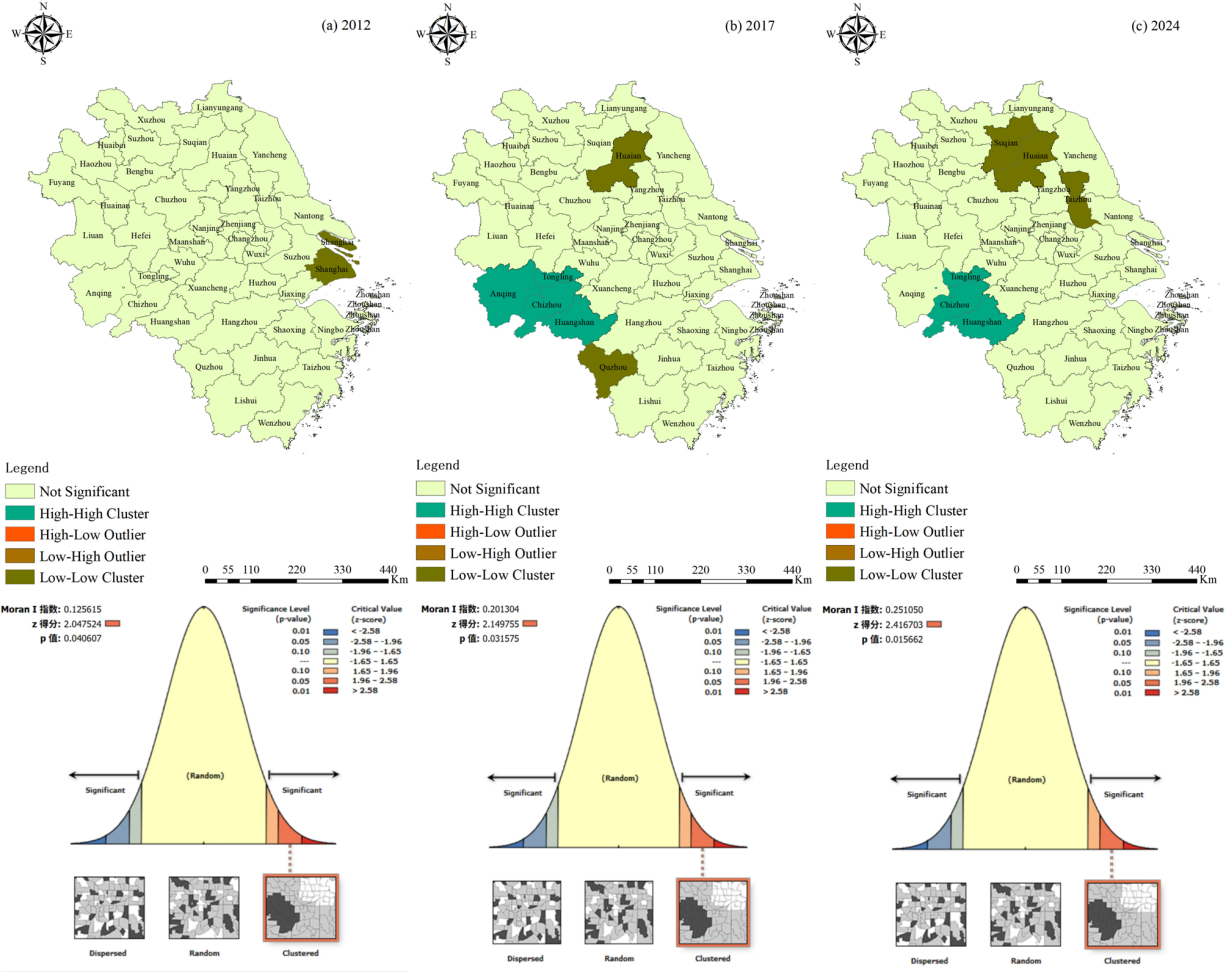
Trends in Spatial Autocorrelation from 2012 to 2024. The map was generated using ArcGIS 10.2. The base map is the standard map (survey number GS(2020)3189) obtained from the Ministry of Natural Resources of China’s Standard Map Service website. URL: http://bzdt.ch.mnr.gov.cn/browse.html?picId=%224o28b0625501ad13015501ad2bfc0421%22.

#### Spatial econometric results

Based on Moran’s I test, which confirms substantial positive spatial dependence in UGT, this study employed three distinct spatial weight matrices: the Economic Distance Matrix (EDM), the Economic Weight Matrix (EWM), and the Economic-Geographic Nested Matrix (EGNM). The LM-error and LM-lag tests were significant at the 5% level for all three matrices, indicating that a spatial econometric model is appropriate. Subsequent tests, including the LR test, the Wald test, and the Hausman test, all rejected the null hypotheses that the Spatial Durbin Model (SDM) should be simplified to either the Spatial Autoregressive Model (SAR) or the Spatial Error Model (SEM). Consequently, the SDM was selected as the preferred model. The spatial econometrics results are outlined in Table 6. The results demonstrate that the log-likelihood is maximized under the dual fixed effects SDM specification for all three matrices, thereby confirming the selection of the dual fixed effects SDM for the primary analysis.

The empirical results reveal that the impact of AI on UGT is characterized by a significant local driving effect, whereas the spatial spillover effect remains weak and inconsistent, thereby validating Hypothesis H2. Specifically, the direct effect of AI remains robustly significant under both individual and dual fixed effects, underscoring its strong supportive role in local green transition. Conversely, the indirect effects are largely insignificant across matrices, with only the EGNM exhibiting a marginal positive spillover under time fixed effects. The findings suggest that economic linkage does not inherently facilitate technology spillovers; rather, a “siphon effect” driven by economic disparities within the YRD may inhibit regional mutualism. Furthermore, the significant impact of AI under the EGNM highlights the critical role of resource endowment and absorptive capacity in technology diffusion. In summary, AI-enabled green transition in the YRD exhibits a pattern of “strong local driving, weak spatial correlation,” suggesting that regional synergy mechanisms require further optimization.

**Table 6:** Spatial econometric regression results and effect decomposition.

Variable	EDM			EWM			EGNM		
	time	ind	both	time	ind	both	time	ind	both
AI	0.097^**^	0.568^***^	0.558^***^	0.242^*^	0.533^***^	0.636	0.064	0.537^***^	0.548^***^
	(2.110)	(3.490)	(3.470)	(1.800)	(3.740)	(1.200)	(0.770)	(3.380)	(3.500)
W×AI	-0.076	-0.161	-0.139^*^	0.273	-0.655^**^	3.882	0.745^*^	0.056	-0.785
	(0.320)	(-0.560)	(-0.420)	(0.040)	(-0.820)	(0.280)	(1.660)	(0.120)	(-1.170)
Controls	Yes	Yes	Yes	Yes	Yes	Yes	Yes	Yes	Yes
Direct Effects	0.100	0.571^***^	0.563^***^	0.242^**^	0.535^***^	0.597^*^	0.051	0.538^***^	0.564^***^
	(1.100)	(3.480)	(3.410)	(2.277)	(3.730)	1.580)	(0.590)	(3.370)	(3.430)
Indirect Effects	-0.084	-0.173	-0.191	0.041	-0.651	1.642	0.583^*^	0.038	-0.771
	(-0.370)	(-0.550)	(-0.570)	(0.010)	(-0.810)	(0.250)	(1.630)	(0.941)	(-1.240)
Total Effects	0.017	0.397^*^	0.372^*^	0.284	-0.115	2.239	0.634^**^	0.575^*^	-0.207
	(0.080)	(1.650)	(1.370)	(0.080)	(-0.150)	(0.320)	(1.940)	(1.340)	(-0.390)
rho	-0.098	-0.021	-0.099	-1.093^**^	-0.071	-1.077^**^	-0.280^**^	-0.012	-0.244^*^
	(-1.180)	(-0.260)	(-1.200)	(-2.030)	(-0.340)	(-2.010)	(-1.920)	(-0.100)	(-1.680)
sigma2_e	0.010^***^	0.008^***^	0.008^***^	0.009^***^	0.009^***^	0.008^***^	0.009^***^	0.008^***^	0.008^***^
	(16.110)	(16.120)	(16.110)	(15.450)	(16.120)	(15.510)	(16.080)	(16.120)	(16.090)
R2	0.087	0.150	0.130	0.148	0.054	0.090	0.125	0.158	0.037
likelihood	471.662	497.376	508.074	479.919	498.266	509.587	475.679	499.498	510.203
N	520	520	520	520	520	520	520	520	520

Note: z-statistics in parentheses. ^*^ *p* < 0.05, ^**^ *p* < 0.01, ^***^ *p* < 0.001.

### Endogeneity test

Endogeneity can lead to biased parameter estimates. To mitigate potential reverse causality between urban green development (UGT) and AI applications, as well as bias arising from omitted variables, this study employs the Two-Stage Least Squares (2SLS) method and Generalized Method of Moments (GMM) for verification. We selected three sets of instrumental variables (IVs) for testing: the interaction term between terrain ruggedness (TR) and digital economy attention (DEA); the interaction term between the number of post offices in 1984 (PON) and the number of internet users (IU); and the one-period lag of the dependent variable (L.UGT). The specific test results are presented in Table 7.

(1) Interaction between the interaction term (TR) and digital economy attention (DEA). This research constructs an instrumental variable (IV) using TR ruggedness, estimated with DEA, to balance geographic exogeneity with temporal dynamics. First-stage regression results show that the coefficient for the interaction term (TR × DEA) is -0.664, and the F-statistic of 7.277 confirms the absence of a weak instrument problem, satisfying the relevance requirement. In the second-stage and GMM estimations, the AI coefficients remain significantly positive at 0.537 and 0.182, respectively, confirming a robust driving effect on UGT after controlling for endogeneity. Furthermore, Sargan and Hansen tests exclude over-identification risks, while the insignificant AR(2) test ensures no second-order serial correlation in the residuals. Consequently, the benchmark findings remain valid after mitigating biases arising from reverse causality and omitted variables.

(2) Interaction between 1984 Post Offices (PON) and Internet Users (IU). This research uses the interaction of the 1984 post office count (PON) and the number of IU as an IV. The historical layout of post offices exhibits technological continuity with modern information technology, while the 1984 data remains strictly exogenous to the current sample period. In the first-stage regression, the coefficient on the interaction term (PON × IU) is 0.031, with an F-statistic of 102.685, well above the threshold for weak instrument identification. The second-stage and GMM estimates yield AI regression coefficients of 0.226 and 0.081, respectively, consistent with the benchmark results. Furthermore, the Sargan tests and AR(2) tests all pass, reaffirming that the conclusion—AI drives urban green transition—remains robust after addressing endogeneity.

(3) One-period Lag of the Dependent Variable (L.UGT). Considering the significant dynamic inertia and path dependence inherent in UGT, this study employs the one-period lag of the dependent variable (L.UGT) as an IV to mitigate omitted-variable bias and isolate inertial effects. The first-stage regression results demonstrate a significant positive impact of L.UGT on current UGT, with an F-statistic of 7.608 confirming the instrument’s adequate explanatory power. In the second-stage regression, the AI coefficient is 1.979 and significant at the 5% level, reaffirming the positive driving effect of AI on UGT. Additionally, the Sargan tests and the AR(2) test verify the validity of the IV and zero second‑order correlation among residuals, ensuring the robustness of the findings within a dynamic panel framework.

**Table 7:** Endogeneity test results.

Variable	(1)			(2)			(3)		
	GMM	First stage	Second stage	GMM	First stage	Second stage	GMM	First stage	Second stage
	UGT	AI	UGT	UGT	AI	UGT	UGT	AI	UGT
L.UGT								0.027^**^	
								(-1.950)	
AI	0.182^**^		0.537^***^	0.081^*^		0.226^***^	0.053		1.979^**^
	(-2.120)		(-2.530)	(-1.150)		(-2.740)	(-0.770)		(-3.310)
TR×DEA		-0.664^**^							
		(-2.130)							
PON×IU					0.031^***^				
					(-7.500)				
Control Variables	Yes	Yes	Yes	Yes	Yes	Yes	Yes	Yes	Yes
Time Fixed Effects	Yes	Yes	Yes	Yes	Yes	Yes	Yes	Yes	Yes
Individual Fixed Effects	Yes	Yes	Yes	Yes	Yes	Yes	Yes	Yes	Yes
Cons	0.962^***^	0.138^***^	0.831^***^	0.958^***^	-0.125^***^	0.916^***^	0.939^***^	0.141^***^	1.195^***^
	(-31.250)	(-3.030)	(-6.840)	(-28.360)	(-10.360)	(-31.560)	(-30.060)	(-3.210)	(-0.680)
Adj-R2		0.928			0.813			0.937	
AR (1)	-2.570^***^			-2.570^***^			-2.530^***^		
AR (2)	0.770			0.780			-0.610		
Sargan test	95.510^**^			95.690^***^			118.110^***^		
Hansen test	36.630			36.910			36.620		
F test	F(1,518) = 7.277^***^			F(1,518) = 102.685^***^			F(1,462) = 7.608^***^		
endogenous	F(1,517) = 0.438(0.928)			F(1,517) = 2.526(0.113)			F(1,461) = 21.945(0.854)		
N	520	520	520	520	520	520	520	520	520

Note: As above.

### Robustness checks

(1) All Variables Lagged by One Period. Drawing on existing literature^50^, the results for all variables lagged by one period are presented in Column (1) of Table 8. The impact of AI on UGT remains significantly positive at the 0.1% level, with a coefficient of 0.583, further confirming the robustness of the benchmark regression results.

(2) Alternative Estimation Models. The Ordinary Least Squares (OLS) and Random Effects (RE) models were employed to replace the benchmark Fixed Effects (FE) model. As illustrated in Columns (2) and (3) of Table 8, the coefficients for AI under OLS and RE specifications are 0.077 and 0.124, respectively. Crucially, the direction of the signs remains unchanged, further confirming that the conclusion that AI promotes the urban green transition does not depend on specific model specifications.

(3) Lagged Explanatory Variable. The core explanatory variable AI was introduced with a one-period lag for regression analysis, with results shown in Column (4) of Table 8. The coefficient for the lagged AI variable is 0.142, which is significantly positive. This further corroborates the reliability of the causal relationship and rules out the interference of contemporaneous instantaneous shocks on the regression results.

(4) Exclusion of Core Cities. Given the potential dominance of major cities, the core cities (Shanghai, Hefei, Suzhou, and Ningbo) within the YRD urban agglomeration were excluded, and the regression procedure was reapplied to the resulting sub‑sample. The results in Column (5) of Table 8 show that the regression coefficient for AI is significantly 0.312. This clarifies that the research conclusions are not driven solely by the performance of a few highly developed cities and are broadly generalizable.

**Table 8:** Robustness check results.

Variable	(1)	(2)	(3)	(4)	(5)
	All variables are lagged by one period	OLS	RE	Lagged AI	Excl. Core Cities
AI	0.583^***^	0.077^***^	0.124^**^	0.142^**^	0.312^**^
	(2.490)	(2.950)	(2.106)	(2.920)	(2.440)
Controls	Yes	Yes	Yes	Yes	Yes
Time FE	Yes	Yes	Yes	Yes	Yes
City FE	Yes	Yes	Yes	Yes	Yes
Cons	0.964^***^	0.958^***^	0.964^***^	1.007^***^	0.997^***^
	(15.840)	(39.140)	(29.470)	(13.710)	(17.750)
R2	0.170	0.150	0.125	0.146	0.241
N	480	520	520	480	468

Note: As above.

### Heterogeneity analysis

#### Heterogeneity of regional endowments

**Fig. 3:**
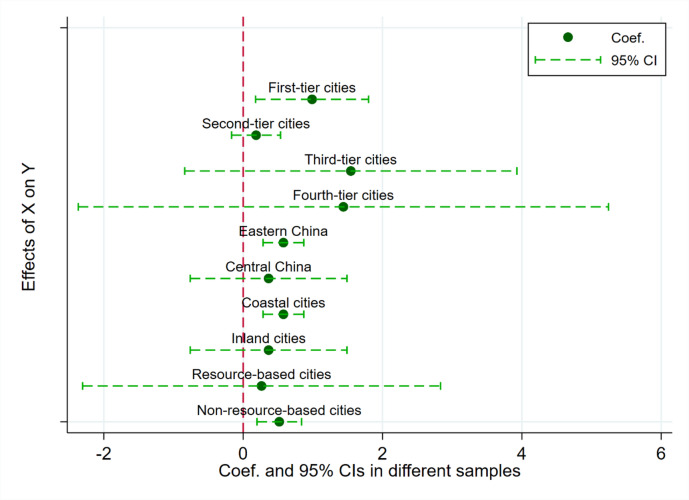
Heterogeneity of regional endowment.

Cities situated in different regions vary significantly regarding resource factors and the extent of socio-economic green development. To examine the heterogeneous effects of AI on UGT across varying regional endowments, subsample regressions were conducted using the aforementioned city-tier classification and categories, such as the National Sustainable Development Plan for Resource-Based Cities (2012–2024). Regressions were run separately for city tier (First- to Fourth-tier cities), geographic placement (Eastern vs. Central China; Coastal vs. Inland cities), and resource dependence (resource-based vs. non-resource-based cities), as presented in Fig. 3. The research findings reveal that the driving effect of AI on UGT is highly heterogeneous. From the perspective of urban hierarchy, only first-tier cities demonstrate a significantly positive and robust promoting effect; conversely, the effects in second-tier cities and below remain statistically insignificant, as stable empowerment pathways have yet to be established. Regarding geographical location, AI exerts a strong and significant impact on the UGT of eastern and coastal cities, buoyed by superior digital infrastructure, whereas central and inland cities are constrained by weaker absorptive capacities. In terms of resource endowment, a clear facilitative effect is observed in non-resource-based cities, while resource-based cities face structural rigidity and path dependence within traditional heavy industrial chains, which impede the rapid penetration of AI technology and render the driving effect insignificant.

#### Proactive government

**Fig. 4:**
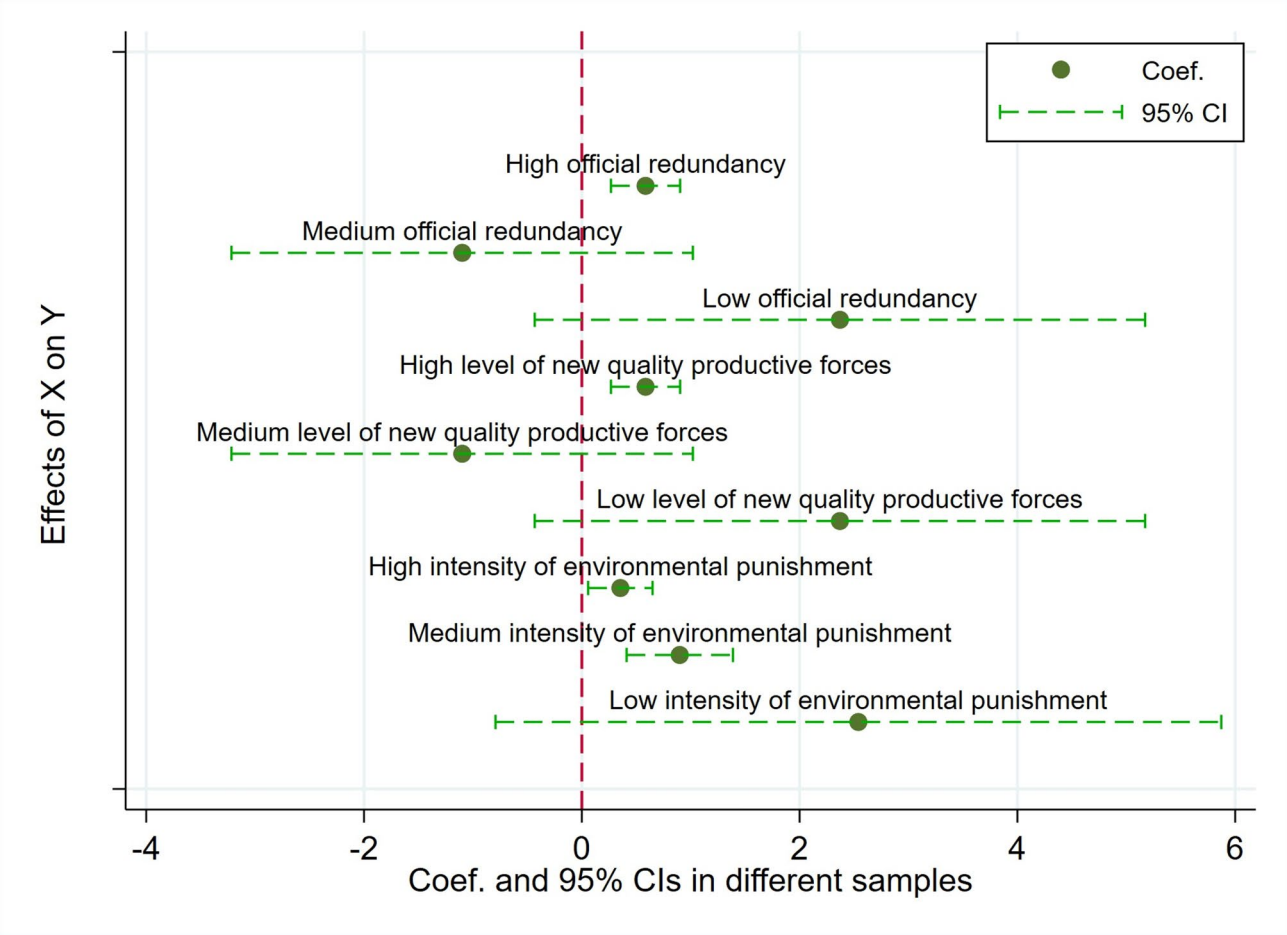
Heterogeneity analysis of proactive government.

The proactive government performs an indispensable function as a booster in promoting the deep integration of AI and UGT. By formulating industrial policies, guaranteeing factor endowments, and reinforcing environmental regulations, the government can effectively correct market failures and create a favorable institutional environment for AI-enabled green development. Following the research methodology of Wang et al.^51–53^, the New Quality Productive Forces Index was measured using the entropy method based on indicators such as the number of employees in emerging industries, employee personal capabilities, infrastructure, future development level, investment in ecological pollution control, carbon trading, and technology R&D. Official Redundancy was proxied by the ratio of public service sector output to the number of employed persons. Environmental Penalty Intensity was measured by the natural logarithm of the number of environmental administrative penalty cases compiled from the Peking University Law database for each region. The full sample was divided into high, medium, and low groups based on the mean value of each indicator for subsample regression, as detailed in Fig. 4. The research findings identify significant conditional heterogeneity in the impact of AI on UGT. Regarding administrative efficiency, a significantly positive effect is observed only in groups with high official redundancy, suggesting that strong administrative execution is a critical pillar for policy implementation. In terms of new, quality productive forces, the effect is significant only in high-level groups, underscoring that advanced digital infrastructure and innovation ecosystems serve as essential thresholds for technology absorption. Regarding environmental regulation, both high- and medium-intensity groups show significant positive effects, confirming that stringent compliance pressure compels enterprises to adopt AI-driven clean technologies. In conclusion, the green empowerment of AI is not universal; its effectiveness is highly contingent upon a city’s institutional execution, factor endowments, and regulatory pressure.

#### Heterogeneity of effective markets

**Fig. 5:**
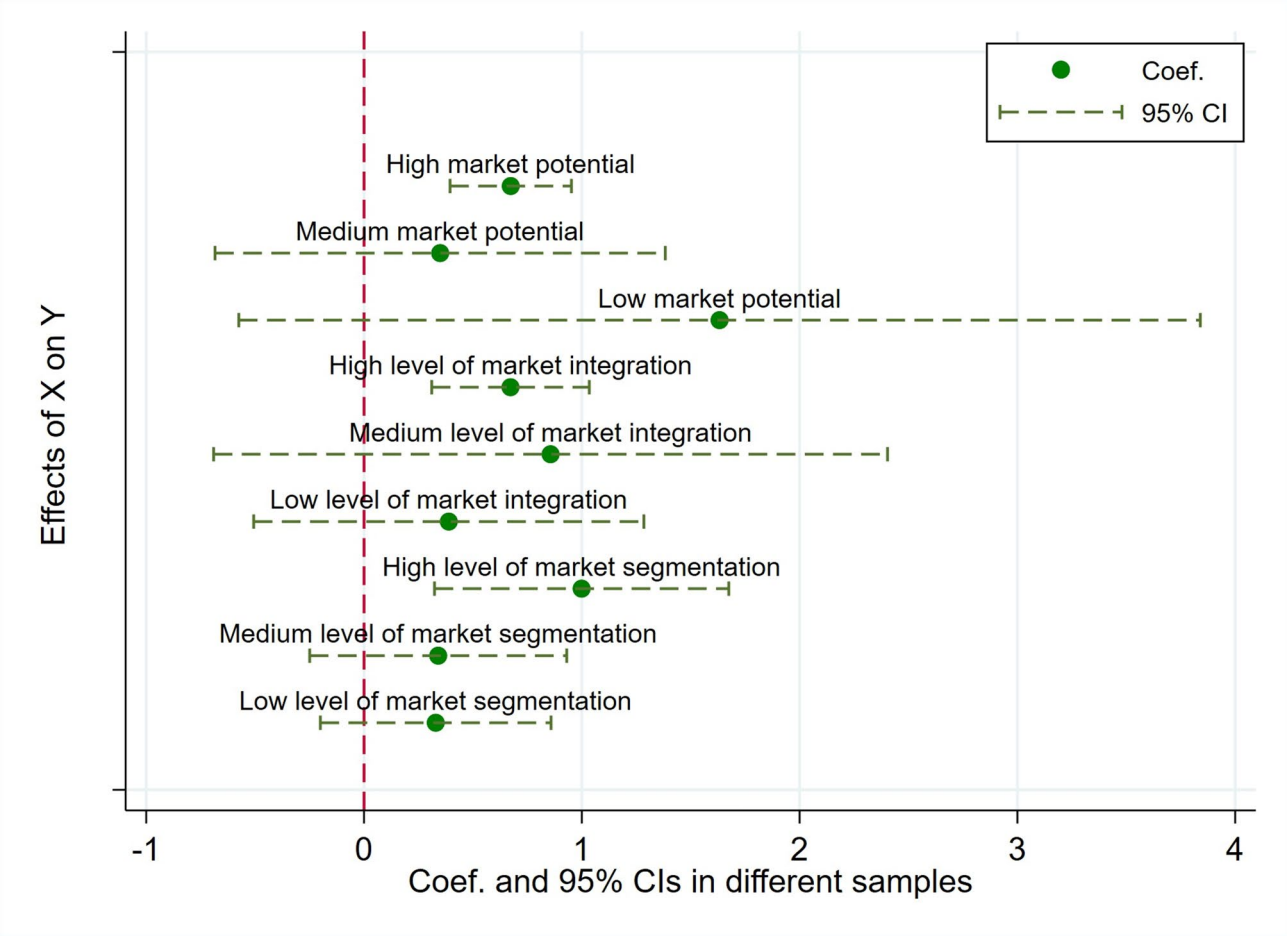
Results of effective market heterogeneity analysis.

Cities with different market environments differ significantly in economic operational efficiency. To uncover the heterogeneous nexus between AI development and UGT across varied market conditions, this study conducts subsample analysis from three dimensions: market potential, market integration, and market segmentation. All samples are classified into high, medium, and low groups based on the mean value of the corresponding indicators. Drawing on Liu et al., Market potential was calculated as the sum of the ratio of city GDP to administrative area and the total GDP density. The market segmentation index was calculated following Yuan’s algorithm^43^, using the mean of the combined variances of relative price fluctuations across seven categories of consumer price indices. Similarly, referring to Peng^54^, the market integration level was measured using the price dispersion index from 2011 to 2024; the results are shown in Fig. 5. The regression results indicate that the enabling effect of AI on UGT exhibits significant heterogeneity, with its positive driving role being particularly pronounced in cities characterized by high market potential, high market integration, and high market segmentation. This divergence stems from the fact that cities with low-to-medium potential struggle to foster stable effects due to insufficient factor agglomeration and weak technical absorption capacity. This heterogeneity aligns with our theoretical predictions, thereby supporting Hypothesis 6, Hypothesis 7, and Hypothesis 8. Meanwhile, low market integration, often coupled with local protectionism, hinders the cross-regional diffusion of technical elements, thereby weakening AI’s promotional impact. Notably, while lower segmentation yields insignificant effects due to limited application intensity, high market segmentation creates local monopolies that “force” enterprises to leverage AI to enhance efficiency and reduce costs. Ultimately, the green dividends of AI are highly contingent on market support and the activation of such forcing mechanisms.

## Mechanism analysis

### Mediation effects

Building on the significance tests presented above, this section further examines the internal mechanisms through which AI influences UGT from the perspectives of production, consumption, and agglomeration. The mediation effects were tested using two-step Structural Equation Modeling (SEM) with Bootstrapping, and the results are displayed in Table 9. Columns (1)–(2) take regional fixed capital stock (RFC) and industrial structure upgrading (ISU) as mediating variables, respectively, to verify Hypotheses H2a and H2b. In Step 1, AI significantly and positively promotes RFC and ISU, indicating that it can effectively drive the accumulation of the fixed capital stock and the upgrading of the industrial structure. The coefficients of RFC and ISU on UGT are significantly negative, while AI still retains a significant positive impact on UGT. Meanwhile, the Bootstrap confidence intervals of the two mediating pathways exclude zero, confirming the existence of valid mediation effects. Given that the direct effect of AI remains significant after incorporating RFC and ISU, both RFC and ISU play full mediating roles. This finding supports Hypothesis H3a and Hypothesis H3b, as AI exerts an inhibitory effect on UGT through two mediating pathways. Column (3) adopts the consumption level (CL) as the mediating variable to test Hypothesis H3c. Step 1 shows that AI significantly increases CL, demonstrating that AI can effectively elevate resident consumption levels. Step 2 indicates that CL exerts a significant positive influence on UGT, whereas the direct effect of AI on UGT becomes insignificant. The corresponding Bootstrap confidence interval also does not contain zero, demonstrating a valid mediation effect. Therefore, consumption level plays a partial mediating role, which supports Hypothesis H2c. This result indicates that AI can indirectly drive urban green transformation by promoting residents’ consumption levels, consistent with the theoretical expectation of Hypothesis H2c.

**Table 9:** Results of mediation effect tests.

Variable	(1)		(2)		(3)	
	Step 1	Step 2	Step 1	Step 2	Step 1	Step 2
	RFC	UGT	ISU	UGT	CL	UGT
AI	1.784^***^	0.089^***^	0.218^***^	0.085^**^	1.538^***^	0.003^**^
	(4.870)	(2.268)	(4.090)	(2.190)	(6.720)	(1.130)
RFC		-0.009^***^				
		(-2.930)				
ISU				-0.055^**^		
				(-2.670)		
CL						0.069^***^
						(2.870)
Cons	8.511^***^	1.029^***^	2.181^***^	1.074^***^	2.660^***^	0.947^***^
	(93.960)	(12.470)	(138.680)	(5.960)	(47.570)	(14.290)
CI	[1.066, 2.503]		[0.113, 0.322]		[0.053, 0.069]	
Conclusion	Full Mediation		Full Mediation		Partial Mediation	

Note: z-statistics in parentheses. ^*^ *p* < 0.05, ^**^ *p* < 0.01, ^***^ *p* < 0.001.

### Moderating effect analysis

The theoretical analysis proposed above suggests that digital infrastructure (DI) and green consumption awareness (GCA) positively affect the promoting role of AI in UGT within the YRD urban agglomeration, while digital industry agglomeration (DIA) has an inverse effect. To verify these hypotheses, a moderating effects analysis was conducted, with the empirical results presented in Table 10. Column (1) of Table 10 presents the results using DI as the moderator. AI and AI×DI both impose a marked positive influence on UGT. Although DI itself harms UGT, the positive and significant interaction effect reveals that DI construction moderates the promoting effect of AI on UGT, validating Hypothesis H4a. This is due to DI being an energy-intensive facility, and integrating it with AI-driven scheduling and optimization can effectively reduce electricity consumption and carbon emissions, thereby facilitating UGT. Column (2) uses the green consumption awareness(GCA) as the moderator, and the results show that both AI and AI×GCA significantly and positively affect UGT. This indicates that the GCA positively moderates the AI’s promoting effect on UGT, thereby validating Hypothesis H4b. This is because the GCA builds a powerful guidance mechanism that incentivizes enterprises to precisely direct AI’s computational advantages toward green product innovation and full-lifecycle carbon management, thereby amplifying the promotional efficiency of AI technology on UGT. Column (3), using digital industry agglomeration (DIA) as the moderator, shows that both AI and DIA lead to marked improvements in UGT, but AI×DIA has a significantly negative impact on UGT. This indicates that DIA inhibits the AI-promoting effect on UGT, validating Hypothesis H4c. This phenomenon likely stems from diminishing marginal returns arising from excessive DIA, which triggers fierce competition for resources and increases environmental carrying capacity, thereby crowding out the green transformation efficiency dividends that AI technology should otherwise deliver.

**Table 10:** Results of moderating effect tests.

Variable	(1)	(2)	(3)
AI	0.861^***^	0.523^**^	0.359^*^
	(2.640)	(2.300)	(1.850)
DI	-0.561^**^		
	(-2.380)		
GCA		-0.066^*^	
		(-1.770)	
DIA			0.053^***^
			(3.350)
AI×DI	0.828^**^		
	(2.230)		
AI×GCA		0.079^**^	
		(2.490)	
AI×DIA			-0.007^**^
			(-2.230)
Controls	Yes	Yes	Yes
Time Fixed Effects	Yes	Yes	Yes
Individual Fixed Effects	Yes	Yes	Yes
Cons	0.933^***^	1.431^***^	0.747^***^
	(21.850)	(3.860)	(6.120)
R2	0.158	1.000	1.000
N	520	520	520

Note: As above.

###  Threshold effect

Considering the potential for dynamic effects in how AI influences UGT, this study applies threshold regression analysis to the sample data to further examine the non-linear characteristics of AI’s impact on UGT. Productive Service Agglomeration (PSA) and Agricultural Agglomeration (AGA) were selected as threshold variables, and sequential tests for single-, double-, and triple-threshold regimes were conducted to identify the optimal threshold quantity. The outcomes are presented in Table 11 (Columns 1 and 2). For the threshold variable of Productive Service Agglomeration (PSA), the double-threshold effect is statistically significant at the conventional level with a threshold value of 0.981. In terms of Agricultural Agglomeration (AGA), the double-threshold effect passes the significance test with remarkable significance. Overall, both PSA and AGA exhibit significant double-threshold characteristics, indicating that the influence of AI on UGT exhibits distinct regime-switching features when the levels of productive service and agricultural agglomeration cross their respective thresholds.

**Table 11:** Results of threshold effect tests.

Threshold Variable	No. of Thresholds	Threshold Value	F-Statistic	P-Value	BS Iterations	Critical Value		
						1%	5%	10%
PSA	Single Threshold	0.981	-310.470	0.803	300	-160	-100	49.465
	Double Threshold	0.981	-174.040	0.003	300	-330	-310	-250
AGA	Single Threshold	15.039	-302.580	0.527	300	-180	-110	-36.51
	Double Threshold	15.039	0.001	0.000	300	-250	-210	-120

Table 12 shows that the threshold value of productive service agglomeration (PSA) is 0.981. The impact of AI on UGT remains positive across both regimes. When the level of productive service agglomeration is lower than the threshold of 0.981, the influence coefficient of AI on UGT is significantly positive at 0.298. By contrast, when PSA reaches or exceeds 0.981, the corresponding coefficient becomes statistically insignificant, which verifies Hypothesis 4a. In terms of agricultural agglomeration (AGA), when the agglomeration level is below the threshold of 15.039, the regression coefficient of AI on UGT is significantly positive at 0.314. When AGA rises to 15.039 or above, the coefficient remains significantly positive at 0.257, though the marginal promoting effect is obviously weakened, thereby validating Hypothesis 4b. Overall, the improvement effect of AI on UGT exhibits evident nonlinear characteristics. As productive service agglomeration and agricultural agglomeration cross their respective threshold levels, the green-promoting effect of AI shows a gradually decaying trend.

**Table 12:** Results of threshold effect regression.

Variable	(1)	(2)
AI	0.347^***^	0.334^***^
	(2.660)	(2.480)
PSA ≥ 0.981	0.121	-
	(1.660)	-
PSA<0.981	0.298^**^	-
	(2.490)	-
AGA ≥ 15.039	-	0.257^**^
	-	(2.270)
AGA<15.039	-	0.314^***^
	-	(19.570)
Control Variables	Yes	Yes
Cons	1.020^***^	1.017^***^
	(21.260)	(22.500)
Time Fixed Effects	Yes	Yes
Individual Fixed Effects	Yes	Yes
N	520	520
R^2^	0.681	0.700

Note: As above.

## Discussion

This study first employed benchmark regression, the SDM with double fixed effects, and the threshold regression model to thoroughly investigate the direct effects, spatial spillover effects, and non-linear characteristics of AI on UGT within the YRD urban agglomeration. Secondly, the intrinsic transmission mechanisms were decomposed using mediation and moderation effect models. Finally, a systematic heterogeneity analysis was conducted from the perspectives of regional endowment, government action, and market environment. The core innovation of this study lies in moving beyond the limitations of previous research, which viewed AI as a linear, homogeneous driving force. We constructed a comprehensive analytical framework that integrates spatial correlation, non-linear thresholding, and multi-dimensional heterogeneity, revealing the complexity of AI-enabled UGT in the YRD urban agglomeration from both overall-effect and localized-segmentation perspectives. This research confirms the prevailing view that AI significantly enhances UGT, supporting the core assertion of the biased technical change theory that technological innovation can reduce environmental costs^21^. Furthermore, the moderating role of digital infrastructure as a positive accelerator is also verified, consistent with existing findings on new digital infrastructure^55^. However, in contrast to some existing studies, this research yields a series of more nuanced and challenging new findings:

First, prior studies often focused on the positive mediating role of fixed capital investment and industrial structure upgrading^56^. However, this study highlights that this relationship exhibits a significant inhibitory effect during the sample period. Conversely, the consumption level is currently the key positive channel driving AI-induced UGT. This implies that simple capital deepening, if not intelligently configured through AI, may trigger an energy rebound effect, offsetting the energy-saving benefits of technology. The key to breaking this deadlock lies in demand-side changes guided by green consumption philosophies.

Second, unlike the view held by some scholars that AI technology has a universal and undifferentiated empowering effect^57,58^, this study reveals a significant resource curse phenomenon. In resource-based cities, the structural rigidity of traditional heavy industry chains creates a strong resistance to AI penetration, preventing AI from acting as an engine of UGT as it does in non-resource-based cities. Moreover, the empowering effect of AI is found to be subject to strict industrial agglomeration thresholds, though the pattern differs from a simple “weak‑to‑strong” leap. Specifically, when productive service agglomeration (PSA) exceeds a critical value, the positive impact of AI on UGT becomes statistically insignificant. For agricultural agglomeration, crossing the threshold leads to a significant but weakened marginal promoting effect. These results indicate that AI’s green driving force does not automatically intensify with higher agglomeration; instead, it exhibits diminishing returns or even complete disappearance beyond optimal agglomeration levels. This non-linear pattern suggests that excessive agglomeration may induce resource crowding and knowledge-spillover inefficiencies, thereby constraining AI’s enabling role in urban green transition.

Third, while Ibe et al. emphasized the universal effectiveness of government intervention, this study finds that the efficacy of proactive government action is conditional on a city’s institutional capacity and technological base^59^. Specifically, the driving effect of AI on UGT is significant only in cities with high administrative execution capacity and medium‑to‑high environmental regulation intensity. Moreover, contrary to the notion that government intervention works best when new quality productive forces are low, our results show that AI’s positive impact is significant only in cities with high levels of new quality productive forces. This suggests that hard government constraints and strong administrative execution are necessary but not sufficient conditions; they must be complemented by a mature local absorptive capacity to translate AI into green dividends. In contrast, cities with low new quality productive forces fail to benefit significantly from AI even under stringent regulations, indicating that technological readiness is a prerequisite for effective government intervention.

Furthermore, the effectiveness of AI in promoting UGT is highly dependent on the market environment. The positive driving effect of AI is strongest in cities characterized by high market potential, high market integration, and high market segmentation. Existing studies have shown that the industrial structure optimization effect of AI appears stronger in locations with advanced market development, thereby enabling a fuller empowerment of green development^60^. By comparison, the promotional influence of the digital economy on green total factor productivity varies substantially across regions, showing stronger performance in eastern areas and non-resource-dependent cities. High market potential provides sufficient demand and factor supplies, thereby lowering the adoption cost of AI^61^. High market integration facilitates the cross‑regional diffusion of technological factors and, through its mediating effect, effectively improves green development efficiency. High market segmentation, by creating local monopolies, “forces” enterprises to adopt AI for internal efficiency gains and cost reduction^62^. Notably, although existing literature suggests that regional market segmentation tends to hinder corporate green technological innovation and carbon neutrality performance, such findings are mainly confined to conventional segmentation contexts. Existing scholarship widely recognizes that regional market fragmentation limits progress in green technology and carbon neutrality, though this rule primarily applies to general contextual conditions. This finding both differs from the conventional view that market segmentation hinders technological innovation and echoes perspectives that market segmentation and competition can amplify the environmental benefits of digital intelligence^63^. In cities lacking these market conditions, the promoting effect of AI becomes weak or insignificant.

Together, these findings suggest that AI’s green-enabling effect is not automatic but a systemic outcome requiring the synergistic support of a proactive government and an effective market. A growing body of research focuses on the synergistic governance mechanism between government and market. Qiu et al. find that the impact of the digital economy on green technology innovation exhibits significant nonlinear threshold effects, and that, under the market-government synergy threshold, the digital economy’s facilitating impact rises steadily^64^. Empirical studies on government-market green governance synergy (GMGG) also show that government-market partnerships play a key role in allocating green resources and facilitating cross-organizational collaboration, with their synergistic effects significantly influencing corporate green innovation, and these effects are moderated by mediating channels such as marketization processes^65^. Moreover, research on the combination of command-based and market-based environmental regulations indicates that dual-pilot policies are more effective than single-pilot policies in balancing environmental protection and economic growth^66^. Therefore, the promotion effect of AI on the green transition can be maximized when the government’s hard execution capacity and the intensity of environmental regulation complement the market’s endogenous demand structure.

## Conclusions and policy implications

###  Conclusions

(1) AI has evolved into a significant driver of UGT within the YRD urban agglomeration. However, this impact exhibits a distinct spatial characteristic of “strong localized driving forces paired with weak spatial correlation.” The study identifies a pronounced regional imbalance and a “tier inversion” phenomenon: the driving effect of artificial intelligence on the urban green transition is evident in clear hierarchical differentiation. Only first-tier cities show a significant positive impact, while the influence in second-, third-, and fourth-tier cities is statistically insignificant, indicating that lower-tier cities lack the capacity to absorb AI dividends to advance UGT. In contrast, first-tier cities face sustained transition pressures due to deep-seated industrial inertia and the inherent complexity of their urban systems. From a spatial evolution perspective, the spatial autocorrelation of UGT in the YRD urban agglomeration has intensified annually, culminating in a “High-High cluster club” centered in southern Anhui. This manifests as a pattern of contiguous linkage rather than isolated polarization. Nevertheless, positive cross-regional spillover effects are currently suppressed by the “siphon effect” stemming from economic disparities. This suggests that resource endowments and technological absorptive capacity remain the critical bottlenecks restricting the diffusion of AI-driven green dividends. Consequently, regional synergy mechanisms require further optimization to dismantle spatial barriers and facilitate cross-boundary integration.

(2) The study identifies significant multi-dimensional heterogeneity in AI’s influence on UGT, contingent upon the synergy between regional endowments, proactive government, and effective markets. Regarding endowments, a robust promoting effect is localized in first-tier, coastal, and non-resource-based cities, while others are constrained by weak absorptive capacity and path dependence. Institutionally, high administrative efficiency, advanced new quality productive forces, and stringent environmental regulations serve as essential thresholds for AI empowerment. Market-wise, the dividends are most pronounced in environments with high potential, integration, and segmentation. Ultimately, AI’s green dividends are not automatic but depend on the strategic alignment of policy enforcement, market maturity, and regional characteristics.

(3) The impact of artificial intelligence on the urban green transition is non-linear and context-dependent. In terms of indirect effects, AI exerts a fully mediating inhibitory effect on the UGT through fixed capital stock and industrial structure upgrading, whereas consumption level plays a partial positive mediating role. Digital infrastructure and green consumption awareness positively moderate AI’s green-promoting effect, whereas excessive agglomeration in the digital industry generates an inverse moderating effect. Both productive service agglomeration and agricultural agglomeration exhibit double-threshold characteristics: once the respective thresholds are exceeded, AI’s green dividends diminish sharply. Therefore, unlocking AI’s sustainable potential requires building a balanced industrial ecosystem that prioritizes qualitative optimization over scale expansion.

### Policy implications

(1) To address path dependence caused by industrial inertia and systemic complexity in first-tier cities, a differentiated empowerment strategy should be implemented. This involves leveraging AI to reconstruct legacy systems, optimize resource allocation, and eliminate systemic lock-in. Conversely, lower-tier cities should harness their green agility and late-mover advantages—stemming from lighter industrial burdens and lower transition costs—to cultivate green competitiveness centered on intelligent manufacturing. To mitigate stagnation in resource flows caused by the siphon effect, it is essential to establish regional technology-sharing pools and mechanisms to compensate for computing power. Such initiatives facilitate flexible cross-regional factor allocation, transitioning from unidirectional resource absorption by central hubs to a paradigm of bilateral regional empowerment, thereby alleviating spatial spillover constraints. Furthermore, to overcome absorptive capacity bottlenecks in peripheral areas, talent decentralization programs and heterogeneous infrastructure support should be deployed to bridge resource gaps and enhance the digestion and secondary development of external green technology spillovers. Finally, by leveraging the strong connectivity of industrial chains, stakeholders should promote green-intelligent supply chain integration to bypass administrative barriers. Integrating dynamic spatial evaluation mechanisms into regional assessments will further dissolve local isolationism, driving a transition from localized driving forces toward systemic regional coupling.

(2) In view of the challenges confronting AI-driven UGT in the YRD urban agglomeration—including unbalanced factor endowments marked by systematic locking-in of first-tier cities and technological absorption bottlenecks in inland and resource-based cities, mismatched institutional support reflected in insufficient administrative execution and misalignment between environmental regulation intensity and industrial foundations, as well as lagging market mechanisms-targeted countermeasures should be adopted. It is essential to implement hierarchical and precise empowerment, guide high-tier cities to advance stock industrial transformation, and help inland and resource-based cities break through technological absorption thresholds; strengthen proactive government guidance by improving administrative efficiency and establishing a differentiated environmental regulation system; optimize the effective market environment by dismantling administrative barriers to release spatial spillover dividends; and build a regional coordination mechanism to drive the transition of the entire region from partial driven development to systematic coupling.

(3) Regarding the complexity of the mechanism and its evolutionary rules, first, the temporary crowding-out risks that AI poses to fixed capital stock and industrial structure upgrading should be carefully assessed to prevent the erosion of green dividends through energy rebound and structural congestion. It is necessary to advance green technological retrofitting and energy-efficiency regulation simultaneously. Second, the positive drivers of resident consumption level, digital infrastructure, and green consumption awareness should be actively leveraged to amplify AI’s green potential through demand‑side incentives and coordinated digital infrastructure development. Third, vigilance is required against diminishing marginal returns and resource competition induced by excessive agglomeration in the digital industry; an early warning mechanism for agglomeration scale should be established to guide its orderly evolution toward green innovation. Finally, the agglomeration levels of productive services and agriculture should be precisely controlled within their respective thresholds to prevent the attenuation of AI’s green effect after the thresholds are crossed.

## Limitations and future research directions

This study has several limitations that suggest directions for future research. First, our analysis focuses exclusively on the Yangtze River Delta urban agglomeration, a highly developed economic hub. Findings such as tier inversion and spatial synergies may not generalize to less developed inland regions or other international contexts. Second, due to data availability, we measure AI development using macro‑level proxy variables and digital economy indices. As emerging technologies such as large language models and generative AI reshape industrial landscapes, future studies should incorporate granular firm‑level data to better isolate the micro‑mechanisms of AI‑driven green transformation. Third, the energy rebound paradox remains a critical concern. Although AI improves operational efficiency, the rising absolute energy demand from data centers and high‑performance computing clusters—implied by the masking effect observed in our analysis—requires rigorous investigation. Future research must assess whether digital advancement can remain compatible with global net‑zero objectives.

## Data Availability

The data that support the findings of this study are available from the corresponding author (W.Y and L.Z) upon reasonable request.DOI: 10.6084/m9.figshare.32149495URL: https://figshare.com/account/items/32149495/edit.
